# FSHD Myotubes with Different Phenotypes Exhibit Distinct Proteomes

**DOI:** 10.1371/journal.pone.0051865

**Published:** 2012-12-18

**Authors:** Alexandra Tassin, Baptiste Leroy, Dalila Laoudj-Chenivesse, Armelle Wauters, Céline Vanderplanck, Marie-Catherine Le Bihan, Frédérique Coppée, Ruddy Wattiez, Alexandra Belayew

**Affiliations:** 1 Laboratory of Molecular Biology, Research Institute for Health Sciences and Technology, University of Mons, Mons, Belgium; 2 Department of Proteomics and Microbiology, Research Institute for Health Sciences and Technology, University of Mons, Mons, Belgium; 3 INSERM U1046 Physiologie et Médecine expérimentale Cœur et Muscle, CHU A. de Villeneuve, Montpellier, France; 4 University Pierre et Marie Curie- Paris 6, UM 76, INSERM U974, CNRS UMR 7215, Institut de Myologie, Paris, France; University of Iowa, United States of America

## Abstract

Facioscapulohumeral muscular dystrophy (FSHD) is a progressive muscle disorder linked to a contraction of the *D4Z4* repeat array in the *4q35* subtelomeric region. This deletion induces epigenetic modifications that affect the expression of several genes located in the vicinity. In each *D4Z4* element, we identified the double homeobox 4 (*DUX4*) gene. *DUX4* expresses a transcription factor that plays a major role in the development of FSHD through the initiation of a large gene dysregulation cascade that causes myogenic differentiation defects, atrophy and reduced response to oxidative stress. Because miRNAs variably affect mRNA expression, proteomic approaches are required to define the dysregulated pathways in FSHD. In this study, we optimized a differential isotope protein labeling (ICPL) method combined with shotgun proteomic analysis using a gel-free system (2DLC-MS/MS) to study FSHD myotubes. Primary CD56^+^ FSHD myoblasts were found to fuse into myotubes presenting various proportions of an atrophic or a disorganized phenotype. To better understand the FSHD myogenic defect, our improved proteomic procedure was used to compare predominantly atrophic or disorganized myotubes to the same matching healthy control. FSHD atrophic myotubes presented decreased structural and contractile muscle components. This phenotype suggests the occurrence of atrophy-associated proteolysis that likely results from the DUX4-mediated gene dysregulation cascade. The skeletal muscle myosin isoforms were decreased while non-muscle myosin complexes were more abundant. In FSHD disorganized myotubes, myosin isoforms were not reduced, and increased proteins were mostly involved in microtubule network organization and myofibrillar remodeling. A common feature of both FSHD myotube phenotypes was the disturbance of several caveolar proteins, such as PTRF and MURC. Taken together, our data suggest changes in trafficking and in the membrane microdomains of FSHD myotubes. Finally, the adjustment of a nuclear fractionation compatible with mass spectrometry allowed us to highlight alterations of proteins involved in mRNA processing and stability.

## Introduction

Facioscapulohumeral muscular dystrophy (FSHD1A: OMIM #158900) is one of the most frequently occurring hereditary muscle disorders. It affects 1/17,000 births and is characterized by progressive muscle weakness, beginning with facial muscles and the shoulder girdle, followed by the pelvic girdle and the muscles of the lower extremities.

FSHD is associated with contractions of the *D4Z4* repeat array in the *4q35* subtelomeric region. In non-affected individuals, this array is comprised of 11–100 tandem copies of the 3.3-kb *D4Z4* element, whereas patients with FSHD only have 1–10 *D4Z4* copies [1], [2]. The molecular mechanism underlying FSHD is not completely clear, but it is commonly accepted that this deletion induces a chromatin remodeling event that could change the expression of several genes in the vicinity of this locus (reviewed in [3]). Normally, the DNA of the *D4Z4* repeat is densely methylated, but in patients with FSHD, hypomethylation has been observed at specific sites in *D4Z4* [4]. In healthy myoblasts, a nuclear matrix attachment site (S/MAR) was found upstream of the repeated elements, which suggests that *D4Z4* and the upstream genes reside in two chromatin loops. This site is weakened in FSHD myoblasts, thus enabling *D4Z4* and the upstream genes to locate into a single loop and allowing *cis*-regulation [5]. A recent study indicated that Polycomb group proteins induced chromatin repression on large *D4Z4* arrays in healthy cells, whereas a long non-coding RNA expressed from the contracted locus recruited the Trithorax group protein Ash1L and promoted histone H3 lysine 36 dimethylation and chromatin opening [6]. Several FSHD candidate genes located centromeric of *D4Z4* have been proposed, including ANT1 (adenine nucleotide translocator 1 gene) [7], *FRG1* (FSHD-related gene 1) [8], *FRG2* [9] and *DUX4c* (double homeobox 4 centromeric) [10].

The *D4Z4* units contain an open reading frame (ORF) with a double homeobox sequence [1] in which we mapped a functional promoter [11], thus defining the *DUX4* gene. This gene has long been referred to as junk DNA, but recent advances have demonstrated its major role in the development of FSHD. Indeed, we have shown that a stable full-length *DUX4* transcript (*DUX4-fl*) is produced from the last *D4Z4* unit in FSHD that further extends to a polyadenylation signal in the flanking *pLAM* region [12]. These findings were confirmed by a study of genetic polymorphisms in hundreds of patients and thousands of non-affected individuals. Stabilization of the *DUX4* mRNA using this polyadenylation site was found to be necessary for the development of FSHD on a contracted *D4Z4* repeat array [13]. The presence of *DUX4-fl* mRNA in FSHD muscle cells was confirmed in other studies [14], [15], and a model of incomplete *DUX4* silencing during development has been proposed for FSHD [15]. The causal role of DUX4 expression also holds for patients with FSHD1B (OMIM #158901) in which chromatin opening at D4Z4 is not associated with repeat array contraction [13]. DUX4 overexpression is toxic in cell culture [16], [17] and in mouse muscles *in vivo*, where DUX4 causes a TP53-dependent myopathy that requires the DUX4 DNA binding domain [18]. The DUX4 protein is a transcription factor that is expressed at a very low level in primary FSHD myoblasts [12], [15]. DUX4 targets a large set of genes, some of which encode other transcription factors such as PITX1 [12]. DUX4 overexpression in mouse C2C12 myoblasts recapitulates key features of the FSHD molecular phenotype, including the repression of MyoD and its target genes, thus leading to diminished myogenic differentiation and the repression of glutathione oxido-reduction pathway components, which consequently results in increased sensitivity to oxidative stress [17]. In human myoblasts, DUX4 overexpression directly targets genes associated with germline and early stem cell development and activates retroelements. It could influence immune responses and muscle differentiation through the induction of the defensin DEFB103 [19]. Additionally, DUX4 activates E3 ubiquitin ligases [19], [20] such as atrogin-1 and MURF1, thus leading to the formation of atrophic myotubes in FSHD [20].

Understanding the pathological cascade leading to FSHD requires the use of transcriptomic and proteomic analyses to identify the molecular dysregulation associated with the disease. Several studies have investigated gene and/or protein expression in FSHD muscle biopsies [12], [21]–[25] (**Table S1**). These studies have collectively highlighted defects in mitochondrial metabolism, the oxidative stress response and myogenic differentiation. Because degeneration, regeneration and inflammation occur in FSHD skeletal muscles, the use of isolated myoblasts to study early disease-related changes should be more informative than the use of muscle biopsies [26]. Moreover, previous studies have shown that dysregulated genes identified in primary myoblast cultures reflected actual changes that occur in muscle fibers. For the present study, we selected a cellular model that was comprehensively characterized by Barro et al. [27]. This group has established a panel of CD56^+^ FSHD primary myoblasts and matched healthy individuals. Under optimized culture conditions without dexamethasone or insulin, the CD56^+^ FSHD primary myoblasts fused and differentiated into myotubes with morphological abnormalities, i.e., thin hypomorphous (atrophic) myotubes or disorganized myotubes with random nuclei distribution. Both phenotypes were found in different proportions in the myotube cultures derived from individual patients with FSHD. Although a myogenic program dysfunction has been previously described in FSHD [22], [26], the molecular basis for the formation of these two myotube types is not known; thus, we wanted to conduct studies to address this knowledge gap. Two transcriptomic studies were previously performed on control and FSHD myotubes [26], [28]. Because it was previously suggested that miRNAs may contribute to the dysregulation in FSHD [26], [29], the present study focused on the FSHD myotube proteome rather than its transcriptome to better characterize the defect in myogenic differentiation and the emergence of two distinct myotube phenotypes. We thus optimized a method that incorporates differential isotope protein labeling (ICPL) and shotgun proteomic analysis (2DLC-MS/MS: two-dimensional liquid chromatography coupled to tandem mass spectrometry) and studied FSHD myotubes obtained by primary myoblast differentiation using the method established by Barro et al. [27]. The optimization of a nuclear enrichment protocol allowed us to detect and efficiently quantify low-abundance proteins in myotubes where cytoskeletal proteins constitute the major protein class. This optimized approach has enabled us to better define the molecular differences between FSHD and control myotubes, as well as between atrophic and disorganized FSHD myotubes.

## Results

### Cell Samples and Analysis Optimization

In the present study, we compared human primary myotubes derived from FSHD muscle and matching healthy control myotubes [27] (**Table S2**). The myoblasts were propagated without dexamethasone (see Material and Methods) and differentiation was induced upon confluence by decreasing the concentration of fetal bovine serum to 2% for 4 days. These conditions were previously shown to cause DUX4 induction [30].

**Figure 1:**
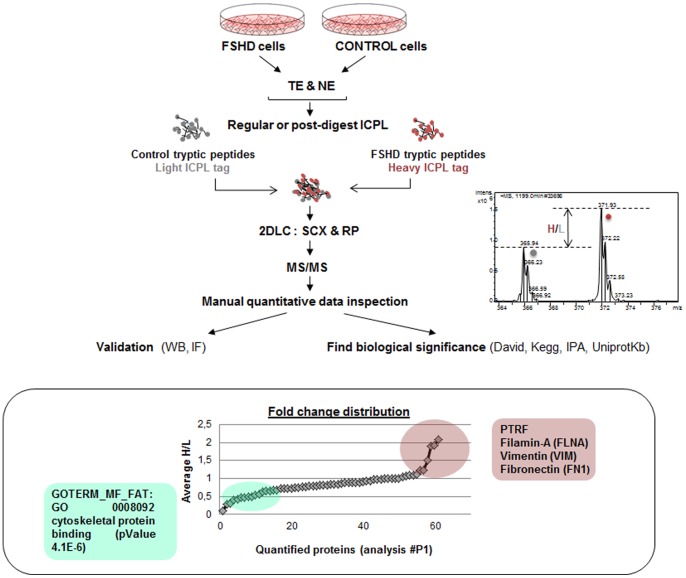
Workflow of the global procedure for protein extraction, labeling and proteomics. FSHD and control primary myoblasts were grown as described (materials and methods) and differentiated into myotubes. Total extracts (TE) and a fraction enriched in nuclear proteins (NE) were analyzed. FSHD and control proteins were submitted to ICPL (Isotope Coded Protein Labeling) before (regular) or after (post-digest) enzymatic digestion with trypsin. FSHD and control samples were labeled with the heavy (H in red) or light (L in grey) ICPL tag, respectively. Equal amounts of FSHD and control labeled peptides were mixed and separated using two-dimensional liquid chromatography (2DLC) using SCX (cation exchange) and RP (reverse phase) columns. Two SCX columns were tested to improve the resolution. The peptides were then analyzed online by MS/MS. The H/L relative peptide quantification ratios were calculated based on the intensities of the peaks in the MS spectra (graph on the right panel). The data were systematically controlled manually, and the quantification data for 3 dysregulated proteins was validated by Western blot. The localizations of these proteins were verified using immunofluorescence. Data interpretation and determination of biological significance in the FSHD context were evaluated using different bioinformatics tools (David database, IPA: Ingenuity Pathway Analysis, Kegg pathway, UniProtKB). The bottom panel represents the fold change distribution of the quantified proteins in preliminary analysis #P1, using regular ICPL and 2DLC-MS/MS on TE of FSHD myotubes from non-affected quadriceps (FSHD8) and a matching control (CTL7), 6 days after differentiation. These cells were previously described in [27], and information relative to these patients is described in **Table S2**. Upregulated and downregulated proteins are circled in red or green, respectively. The most-represented functional categories were determined from the David database (Functional Annotation Charts and Clustering) using Gene Ontology (GOTERM_CC_FAT: Cellular Component, GOTERM_BP_FAT: Biological Process, GOTERM_MF_FAT: Molecular Function). GO annotation and accession numbers are indicated. The p-value is equivalent to the EASE score, which uses a conservative adjustment of Fisher’s exact probability and was applied to identify significantly enriched gene categories.

The procedure for protein extraction, trypsinolysis, tryptic peptide labeling (ICPL: Isotope Coded Protein Labeling), gel-free separation and identification by mass spectrometry (2DLC-MS/MS) was improved at different levels and resulted in an increased number of identified and quantified myotube proteins (**Fig. 1**). Protein quantification was based on the ICPL methodology, which consisted of labeling protein or peptide free amino groups (N-terminal and lysines) with amine-specific reagents containing different stable isotopes. In the present study, FSHD proteins were labeled with the heavy ICPL tag (H) and control proteins were labeled with the light ICPL tag (L). Relative peptide quantification (fold change) was determined from the H/L intensity ratio, and the data were manually validated.

The preliminary experiments that were performed during the optimization steps are reported in **Table S2B**. In the first experiment (**analysis #P1, Table S5**), only 146 proteins were identified, and 61 proteins were quantified. Most of the proteins were ribonucleoproteins and cytoskeletal proteins. Despite a limited number of quantified proteins, the few observed changes reflected FSHD characteristics (**Fig. 1**). Proteins with decreased relative abundance were associated with the cytoskeleton (i.e., MYH2, MYH8, CAP2, transgelin and filamin C), which is indicative of perturbed myogenic differentiation [22], [26]. Three proteins had an H/L ratio higher than 1.5 in FSHD myotubes: the extra-cellular matrix protein fibronectin, the caveolar protein PTRF and the intermediate filament vimentin, as previously described in [31].

### Atrophic and Disorganized Myotubes: A Comparative Proteomic Analysis

**Figure 2:**
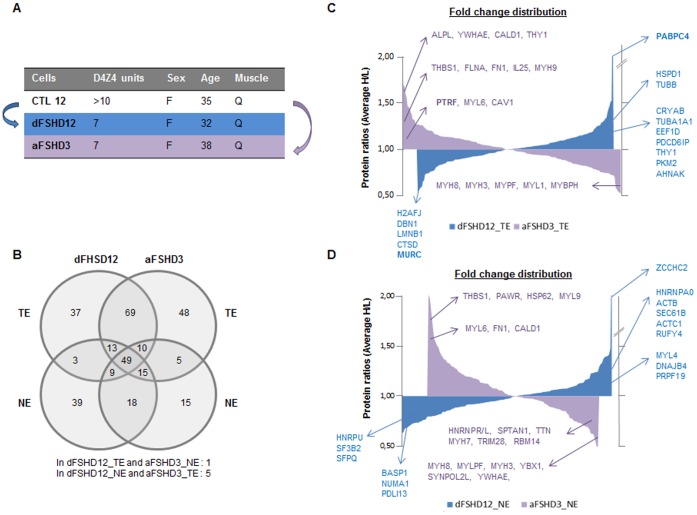
Comparison of quantified proteins in atrophic and disorganized FSHD myotubes. (**A**) Experimental strategy. The proteomes of predominantly atrophic (aFSHD3) or disorganized (dFSHD12) myotubes were compared in two pairs (arrows) with one matching healthy control (CTL12) using post-digest ICPL and 2DLC-MS/MS. The number of *D4Z4* units, sex and age of the patients are indicated, in addition to the biopsy site. F: female, Q: quadriceps. In addition, another matching healthy control (CTL7) was compared to CTL12. (**B**) Venn diagram presenting the number of quantified proteins in TE and NE of atrophic (aFSHD3) or disorganized (dFSHD12) FSHD myotubes and their degree of overlap. (**C**) Fold change distribution of quantified proteins in total extracts of disorganized (blue: dFSHD12_TE) or atrophic myotubes (purple: aFSHD3_TE). (**D**) Fold change distribution of quantified proteins in NE of disorganized (blue: dFSHD12_NE) or atrophic myotubes (purple: aFSHD3_NE). The most dysregulated proteins in each analysis are indicated by the corresponding Hugo Gene symbol. The proteins that are representative of the most dysregulated categories (DAVID database, Functional Annotation Charts, Gene Ontology: GOTERM_BP_FAT) are also shown.

After optimization of the procedure (**Texts S1–3**), we compared the proteomes of predominantly atrophic (aFSHD3) myotubes with those of disorganized (dFSHD12) myotubes. The aFSHD3 and dFSHD12 myoblasts were derived from the non-affected quadriceps of two women with FSHD who had 7 *D4Z4* units and were of similar age. Both myotube types were compared in two pairs with the same matching control (CTL12, **Fig. 2A**). For each sample, total extracts (**#aFSHD3_TE** and #**dFSHD12_TE**) and a fraction enriched in nuclear proteins (hereafter referred to as “nuclear extract”) (**#aFSHD3_NE** and #**dFSHD12_NE**) were analyzed (**Table S7–10**). In addition, to evaluate inter-control protein variations, CTL12 was compared to the myotube total extract from another healthy individual (**#CTL7_TE; Table S11**).

The analysis of aFSHD3 and dFSHD12 myotubes allowed the identification of 503 non-redundant proteins, among which 336 were quantified. The Venn diagram in **Fig. 2B** represents the quantified proteins in individual runs. Nuclear enrichment enhanced the detection of nuclear proteins and increased the total number of quantified proteins by reducing the number of cytoskeletal proteins (**Text S2, Table S6 and Fig. S1**). However, some cytoskeletal proteins were still detectable in the nuclear fraction because of their abundance, their interaction with nucleus components or because of a secondary function in the nucleus, as described for actin and myosin [32]–[35].

**Figure 3:**
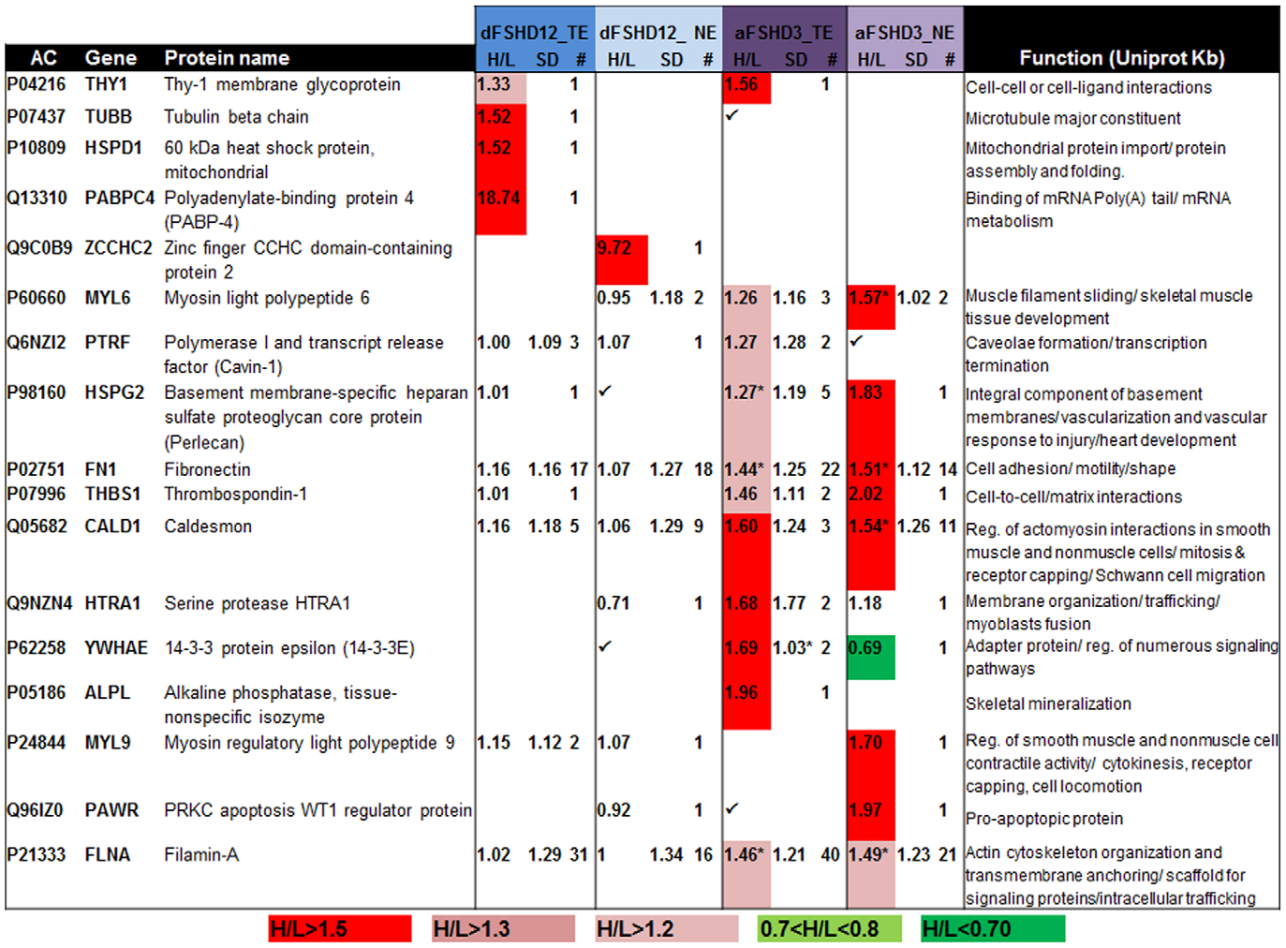
Proteins with increased abundance in atrophic (aFSHD3) or disorganized (dFSHD12) FSHD myotubes as determined by post-digest ICPL and 2DLC-MS/MS. AC: UniProt accession number; Hugo Gene symbol; Protein name; H/L: fold change (✓: identified protein without quantification); SD: geometric standard deviation; #: number of peptides used for quantification; *: statistical significance (p<0.05) determined by Student’s *t*-test. The considered threshold limit is 1.5. Proteins with an H/L ratio greater than 1.5 are highlighted in red; those with a ratio greater than 1.3 are in pink and those with a ratio greater than 1.2 are in light pink. Proteins with an H/L ratio less than 0.7 are highlighted in green and those with an H/L ratio of 0.7 - 0.8 are highlighted in light green. TE: total extract; NE: nuclear extract.

The fold change distributions from each analysis are represented in **Fig 2C-D**. The most dysregulated proteins, as well as proteins that are representative of the most dysregulated categories (DAVID Functional Annotation Chart, Gene Ontology: goterm_bp_fat, p<0.05), are indicated using the corresponding Hugo Gene symbol (**Table S3**). The upregulated proteins with H/L ratios above 1.5 are reported in **Fig. 3** **.**

### The Relative Abundance of Structural Muscle Components is Strongly Decreased in Atrophic Myotubes

In aFSHD3 myotubes, 19% and 12% of the quantified proteins presented decreased or increased abundance relative to the control myotubes, respectively.

In the total (aFSHD3_TE, **Fig. 2C** **, purple**) and nuclear extracts (aFSHD3_NE, **Fig. 2D** **, purple**), the proteins with decreased relative abundance were mostly muscle structural components that are involved in contraction, e.g., myosin heavy chains (MYH8, MYH3, and MYH7), myosin light chains (MYL1 and MYL6B), Titin (TTN) or proteins involved in striated muscle development (MYLPF: myosin regulatory light chain 2 and XIRP1: xin actin-binding repeat-containing protein 1). A minor protein group involved in mRNA splicing was decreased in the nuclear extracts (YBX: nuclease-sensitive element-binding protein 1; HNRNPR: heterogeneous nuclear ribonucleoprotein R/L; and RBM14: RNA-binding protein 14).

The proteins with increased relative abundance in the total extracts (aFSHD3_TE, **Fig. 2C** **, purple**) were involved in cytoskeletal organization (CALD1: caldesmon; THY1: Thy-1 membrane glycoprotein; FLNA: filamin-A; MYH9 and EHD2: EH domain-containing protein 2) and cell motility (YWHAE: 14-3-3 protein epsilon; THBS1: thrombospondin-1; FN1: fibronectin; and MYH9). Some of these proteins are also involved in angiogenesis and cell adhesion (MYH9, FN1, THBS1, and THY1). Intriguingly, the serine protease HTRA1 (aFSHD3 myotubes; **Fig. 3**), was quantified by two peptides with very different H/L ratios, which likely indicates the presence of two isoforms (as described [36]), among which only one was increased in atrophic FSHD myotubes (**Fig. S4**).

In nuclear extracts (aFSHD3_NE, **Fig. 2D** **, purple**), proteins with increased relative abundance belonged to similar categories: actin-filament-based processes (MYL9, MYL6, CALD1, FLNA, MYH9, and LIMA1: LIM domain and actin-binding protein 1) and cell adhesion (THBS1, HSPG2: basement membrane-specific heparan sulfate proteoglycan core protein, and MYH9). The PRKC apoptosis WT1 regulator protein (PAWR) was increased in the nuclear compartment, which is a location that is associated with its pro-apoptotic activity. Surprisingly, the relative abundance of 14-3-3 protein epsilon (YWHAE) was decreased in nuclear extracts (H/L = 0.69) but increased in total extracts (H/L = 1.69) (**Fig. S3**). The 14-3-3 protein family regulates the cell cycle and apoptosis by controlling the nuclear and cytoplasmic distribution of the signaling molecules with which they interact. This observation suggests a dysregulation of the shuttling of 14-3-3ε between the cytoplasm and the nucleus in FSHD myotubes. The intermediate filament component vimentin, which is the homologue of desmin that is expressed in non-differentiated muscle cells, did not present a much higher relative abundance in aFSHD3 myotubes (H/L ratio in aFSHD3_TE: 1.21, H/L ratio in aFSHD3_NE: 1.39); however, a higher H/L ratio (1.90) was observed in a preliminary analysis (analysis #P1**,** **Fig. 1** **and Text S1**).

In summary, atrophic myotubes were mostly characterized by a decreased relative abundance of contractile muscle components and an increased relative abundance of proteins with roles in cell motility and actin cytoskeleton remodeling. Finally, the nuclear fraction was characterized by a decreased abundance of proteins involved in RNA splicing.

### Disorganized Myotubes Exhibit a Higher Relative Abundance of Microtubule Network Regulators

In dFSHD12 myotubes, approximately 13% of the quantified proteins had an H/L ratio lower than 0.8 and 7% had an H/L ratio higher than 1.3. Globally, as compared to atrophic myotubes, the changes were moderate and the dysregulated proteins were involved in various biological processes, with a maximum of only 4 proteins per functional category (**Table S3**).

In total extracts, the proteins with the greatest decrease in relative abundance were the caveolar muscle-related coiled-coil protein (MURC, H/L: 0.53), the lysosomal protease cathepsin D (CTSD; H/L: 0.56), lamin-B1 (LMNB1; H/L: 0.57), drebrin (DBN1; H/L: 0.59) and the histones H2A.J (H2AJF; H/L: 0.59) and H4 (HIST1H4A; H/L: 0.74*) (dFSHD12_TE, **Fig. 2C** **, blue**). The proteins with increased relative abundance may be involved in the morphological disorganization. Indeed, they are mostly involved in cytoskeleton organization and protein complex assembly/biogenesis, e.g., tubulin α-1A chain (TUBA1A), tubulin β chain (TUBB), αB-crystallin (CRYAB), the 60-kDa heat shock protein (HSPD1) and thy-1 membrane glycoprotein (THY1). Although the TUBA1A quantification was supported by 3 peptides (dFSHD12_TE analysis), it should be mentioned that one of these peptides presented an H/L ratio of 1.35 in a study comparing myotubes from two healthy individuals (CTL7 *vs.* CTL12) with the same method (analysis #CTL7_TE**, Table S11**). Proteins with increased relative abundance are also involved in other biological processes such as apoptosis, e.g., programmed cell death 6-interacting protein (PDCD6IP and HSPD1) and immune response-activating signal transduction (THY1 and HSPD1). The most upregulated protein is polyadenylate-binding protein 4 (PABPC4), which is involved in mRNA stabilization and has an 18-fold up-regulation that was validated by Western blot (**Text S4 and** **Fig. S2**).

The nuclear enrichment allowed us to detect additional dysregulated categories of proteins (dFSHD12_NE, **Fig. 2D** **, blue**). The proteins with decreased relative abundance were mostly associated with mRNA splicing, e.g., heterogeneous nuclear ribonucleoprotein U (HNRNPU), splicing factor 3B (SF3B2) and splicing factor proline- and glutamine-rich (SFPQ). Other ones were associated with cytoskeletal organization, i.e., PDZ and LIM domain protein 3 (PDLI13). Three of the proteins with increased relative abundance were structural components of muscle (ACTC1: actin alpha-cardiac muscle 1; ACTB: beta-actin and MYL4: myosin light chain 4), and the other ones were involved in various processes (DNAJB4: heat shock 40 kDa protein 1 homolog; SEC61B: protein transport protein Sec61 subunit beta and HNRNPA0: heterogeneous nuclear ribonucleoprotein A0). The most upregulated protein was ZCCHC2 (zinc finger CCHC domain-containing protein 2), which had a 10-fold increase in relative abundance in FSHD compared to the control sample.

In summary, disorganized myotubes exhibit globally moderate and heterogeneous dysregulation. When compared with atrophic myotubes, myosin isoforms did not have decreased relative abundance. However, the up-regulated proteins were associated with the cytoskeleton, particularly with the regulation of the microtubule network organization. In nuclear extracts, as observed for atrophic myotubes, proteins with decreased relative abundance were involved in RNA splicing, which suggests that this perturbation is a common feature of FSHD myotubes.

### Myosin Isoforms are Differentially Expressed in Atrophic and Disorganized Myotubes

**Figure 4:**
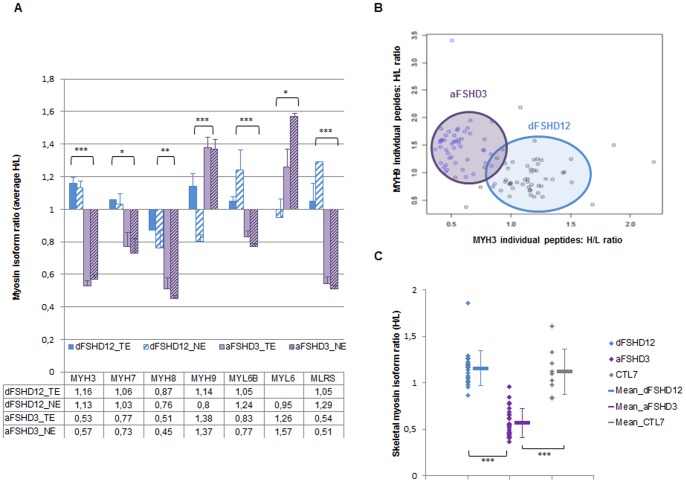
Quantification of myosin isoforms in atrophic or disorganized FSHD myotubes. (**A**) Histogram depicting the relative quantification (H/L ratio) of myosin heavy chains (MYH), myosin light chains (MYL) and myosin regulatory light chains (MLRS) determined by post-digest ICPL coupled to 2DLC-MS/MS analysis of TE and NE derived from atrophic (aFSHD3) or disorganized (dFSHD12) FSHD myotubes. The bottom table indicates the average H/L ratio for each myosin isoform, which is reported on the Y axis. The significance was evaluated using an ANOVA test and a multiple comparison of means (Tukey contrasts) using the R Foundation for Statistical Computing version 2.14.0 software. p<0.001 (***); p<0.01 (**) and p<0.05 (*) were considered significant. (**B**) Principal component analysis with quantified MYH9 and MYH3 peptides in TE and NE. Peptides from atrophic myotubes are represented by blue dots and peptides from disorganized myotubes by black dots. The quantification of these myosin isoforms allows the discrimination of two groups in the scatter plot: one group corresponding to aFSHD3 (underlined in purple) myotubes and the other corresponding to dFSHD12 (underlined in blue) myotubes. The proteomic quantitative data related to the myosin isoforms are presented in **Table S4A,** and bibliographic information about the role of each isoform is given in **Table S4B and S4C**. (**C**) Scatter plot graph representing the H/L ratio corresponding to quantified peptides of skeletal myosin isoforms (MYH3, MYH7, MYH8, MLRS) in analyses dFSHD12_TE, aFSHD3_TE and CTL7_TE. The analysis to the right was conducted as a control for the variability among myotubes from two healthy individuals.

Atrophic myotubes (aFSHD3) are characterized by a drastic decrease in the relative abundance of structural muscle proteins. Whereas myosin heavy (MYH) and light (MYL) chains are largely unchanged in disorganized myotubes (dFSHD12), they present clear perturbations in aFSHD3 myotubes **(** **Fig. 4** **, Table S4)**. The histogram of the average H/L ratios of all myosin isoform peptides from aFSHD3 and dFSHD12 myotubes is a mirror image centered on a H/L value of 1 (**Fig. 4A**). The H/L ratio, MASCOT score, number of quantified peptides and standard deviations are reported in **Table S4A**, and the functions of each myosin isoform are summarized in **Tables S4B and S4C**. In aFSHD3 myotubes, the skeletal muscle myosin isoforms MYH7, MYH3 and MYH8 which are typical of fetal development and muscle regeneration had a significantly lower relative abundance. MYH2, which is the skeletal muscle isoform IIa of myosin, had the same trend for decreased relative abundance as the skeletal muscle regulatory light chain MLRS. In contrast to other myosin isoforms, the so-called “non-muscle myosin” MYH9 (NMHC-A) showed increased relative abundance in atrophic FSHD myotubes, as were the MYL6, MYL9 and MYL12B light chains. Interestingly, preferential interactions were described between MYL12A, MYL12B and MYL9 and non-myosin heavy chains or the essential light chain MYL6 [37]. These interactions are crucial for NMHC integrity. MYH3 and 9 were quantified by 20 and 15 specific peptides, respectively, and the H/L ratio for each peptide was able to discriminate the myotube phenotype as shown by the scatter plot in **Fig. 4B**. As a control, the degree of skeletal myosin (MYH3, -7, -8 and MLRS) peptide variability among myotubes from two healthy individuals (CTL7 *vs.* CTL12) was evaluated using the same method (analysis #CTL7_TE**, Table S11)** and was found to be significantly lower than the degree of variation between aFSHD3 and CTL12 (analysis #aFSHD3_TE, **Fig. 4C**). Taken together, these results indicate a downregulation of myosin isoforms that are characteristic of skeletal muscle in FSHD atrophic myotubes and a switch in favor of non-muscle myosin complexes.

### Caveolar Proteins are Dysregulated in FSHD Myotubes

**Figure 5:**
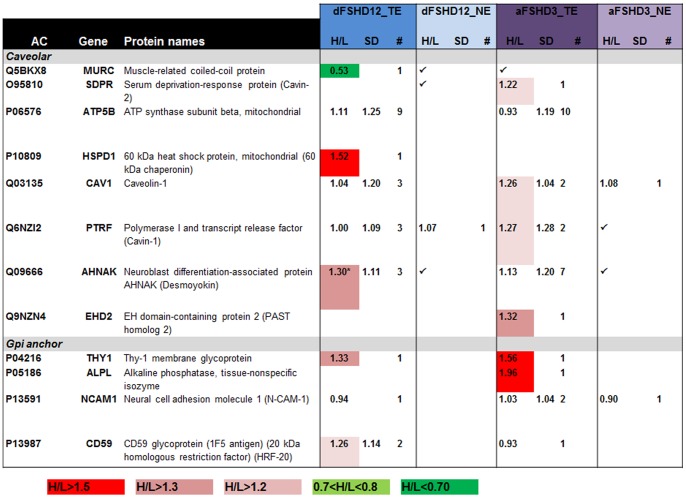
Caveolar and Gpi anchor proteins for which a change in abundance was observed in FSHD myotubes using post-digest ICPL coupled to LC-MS/MS. AC: UniProt accession number; Hugo Gene symbol; Protein name; H/L: fold change (✓: identified protein without quantification); SD: geometric standard deviation; #: number of peptides used for quantification; *: statistical significance (p<0.05) determined by Student’s *t*-test. Proteins with an H/L ratio greater than 1.5 are highlighted in red; those with a ratio greater than 1.3 are in pink and those with a ratio greater than 1.2 are in light pink. Proteins with an H/L ratio less than 0.7 are highlighted in green and those with an H/L ratio of 0.7 - 0.8 are highlighted in light green. TE: total extract; NE: nuclear extract.

Atrophic and disorganized myotubes both exhibited dysregulation of caveolar proteins **(** **Fig. 5** **)**, which comprise a subclass of lipid membrane microdomains that play a major role in signal transduction. Cavins, which are critical for caveolae formation and dynamics [38], were among the most dysregulated proteins. Indeed, the relative abundance of PTRF (polymerase 1 and transcript release factor/cavin-1) was increased 2-fold in a preliminary analysis (analysis #P1**,** **Fig. 1**, **Table S5**) and was moderately induced in analysis aFSHD3_TE (**Fig. 2C**). The relative abundance of MURC (also named cavin-4) was decreased by half in disorganized myotubes (dFSHD12_TE; **Fig. 2C**). Caveolae contain clusters of Gpi-anchor proteins such as alkaline phosphatase and THY-1 membrane glycoprotein. The relative abundance of these proteins was increased in FSHD atrophic myotubes (aFSHD3_TE; **Fig. 2C**). Finally, the extracellular protein AHNAK, which is a member of the dysferlin-protein complex, also had a slightly increased relative abundance in disorganized myotubes (dFSHD12_TE; **Fig. 2C**).

**Figure 6:**
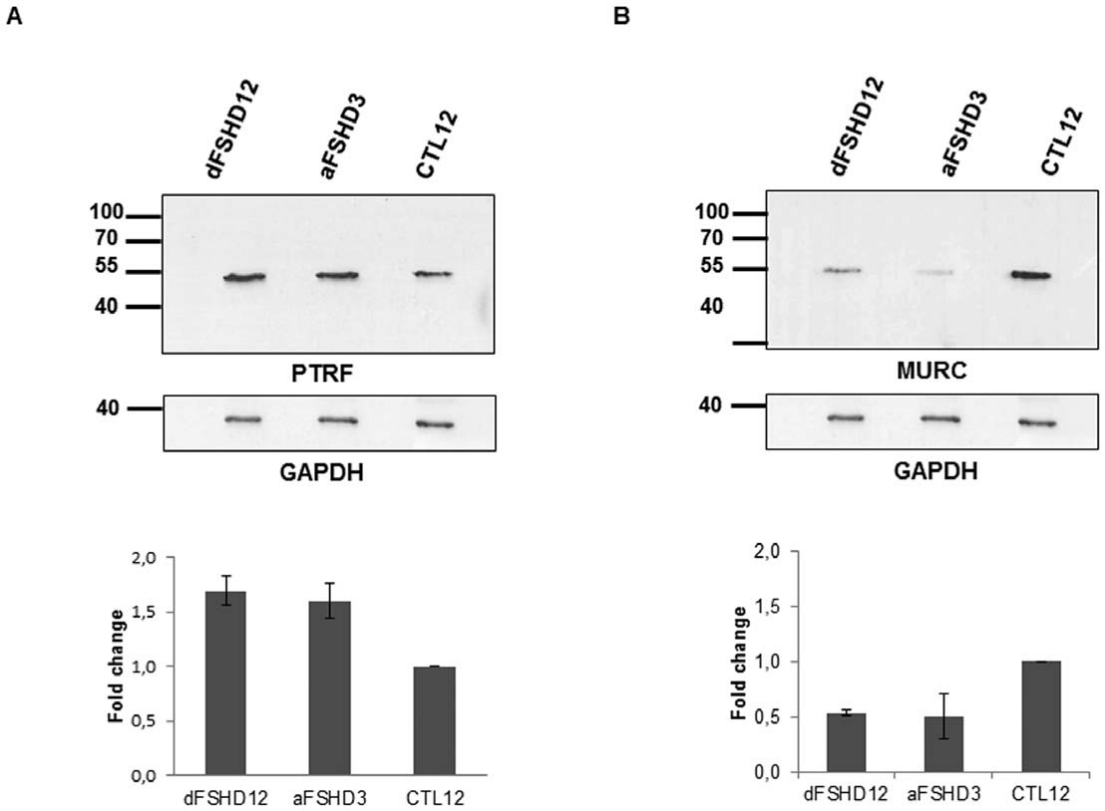
Western blot immunodetection of PTRF and MURC in FSHD and control myotubes. 25 µg of TE from primary myotubes were separated by 12% SDS-PAGE and transferred to a nitrocellulose membrane for Western blotting. PTRF (**A**) and MURC (**B**) were immunodetected with specific antibodies as described in the Materials and Methods section. GAPDH was used as a loading control. aFSHD3 and dFSHD12 are FSHD myotubes that are predominantly atrophic or disorganized, respectively, and were compared to matching control myotubes (CTL12). The bottom panels present the densitometric analysis of biological replicates (n = 2).

**Figure 7:**
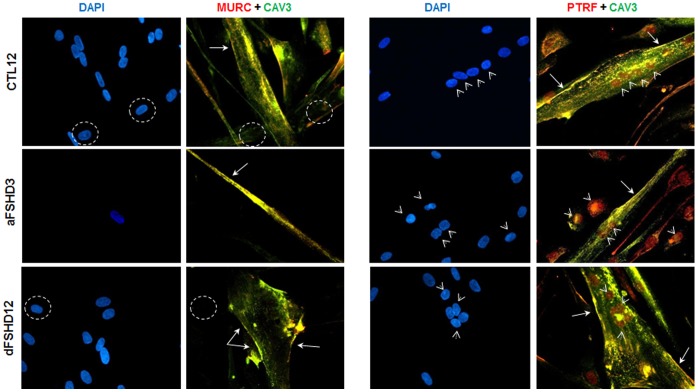
MURC and PTRF detection by immunofluorescence in FSHD and control myotubes. MURC (right panel, red), PTRF (left panel, red) and caveolin-3 (CAV3, green) were detected by co-immunofluorescence in primary FSHD (dFSHD12 and aFSHD3) and control (CTL12) myotubes following 4 days of differentiation. aFSHD3 and dFSHD12 myotubes are predominantly atrophic or disorganized, respectively. The 3 proteins were detected with specific antibodies as described in the Materials and Methods section. DAPI was used to visualize nuclei. The merged signal of CAV3 and PTRF or MURC, representing co-localization, is in yellow. PTRF or MURC staining at the plasma membrane is indicated by arrows and nuclear PTRF staining is indicated by arrowheads. Non-fused myoblasts had weaker MURC staining than myotubes (dotted circles).

As a validation of the quantitative proteomic data, we evaluated PTRF and MURC protein expression by immunodetection on Western blot (**Fig. 6**). The intracellular distribution of PTRF and MURC was assessed by co-immunofluorescence with caveolin-3 (CAV3), which is the major caveolar protein in skeletal muscle, in aFSHD3 and dFSHD12 myotubes after 4 days of differentiation (**Fig. 7**).

PTRF regulates ribosomal RNA transcription and also plays a critical role in the formation of caveolae, stabilization of caveolins and membrane repair. As expected, 55-kDa PTRF was detected in TE of FSHD and control myotubes (**Fig. 6**). In agreement with the proteomic data, densitometry analysis showed that the relative abundance of PTRF was increased 1.6- and 1.7-fold in aFSHD3 and dFSHD12, respectively, as compared to the matched control (CTL12). PTRF was detected by immunofluorescence in the cytoplasm and plasma membrane of myotubes, where it co-localized with CAV3 (**Fig. 7**). As expected because of its role in transcription regulation, PTRF was also detected in myotube nuclei. This distribution did not appear modified in FSHD myotubes.

MURC, which was originally observed at the Z-line of the sarcomere, was also found in muscle cell caveolae. In addition to its association with cardiac dysfunction, MURC is involved in the regulation of skeletal myogenesis, and its expression is perturbed in muscle diseases that are associated with caveolin-3 mutations. MURC was immunodetected after Western blotting in the TE of FSHD and control myotubes (**Fig. 6** **)**, and in agreement with the proteomic data, densitometry analysis indicated a 0.5-fold reduction in its relative expression in dFSHD12 and aFSHD3 myotubes. Immunofluorescence (**Fig. 7**) revealed that MURC co-localized with CAV-3 and that myotubes exhibited a stronger MURC signal than elongated myoblasts, which suggests the induction of MURC upon differentiation, as previously described in C2C12 cells [39]. Similarly to PTRF, the subcellular localization pattern of MURC was not altered in FSHD myotubes.

## Discussion

### Shotgun Proteomics is an Effective Approach for the Analysis of FSHD Myotubes

In the present study, we optimized a method of differential isotope protein labeling (ICPL) and shotgun proteomic analysis (2DLC-MS/MS) to study FSHD myotubes expressing DUX4 [30] and presenting characteristic morphological abnormalities [27]. As observed in our preliminary analysis (**Texts S1–3**), the main disadvantage of the proteomic analysis of myotubes is the abundance of contractile and other cytoskeletal proteins that could mask the detection of lower abundance proteins. The combination of higher-resolution LC separation, post-digest ICPL and nuclear enrichment allowed us to increase the number of quantified proteins. Our data are in agreement with perturbations previously observed in FSHD, and the quantification of 3 dysregulated proteins (PABP4, PTRF and MURC) was confirmed by complementary approaches.

### The Atrophic Myotube Phenotype in FSHD Could be Attributed to DUX4

Under optimized culture conditions without dexamethasone or insulin, CD56^+^ FSHD myoblasts fused to form atrophic or disorganized myotubes. Both phenotypes were present at different proportions in a culture derived from a given FSHD muscle [27], but the molecular basis of these phenotypes is not clearly known. To better understand the defect in the myogenic program previously described in FSHD [22], [26] and the emergence of two myotube phenotypes in FSHD primary culture, the optimized proteomic procedure was used to evaluate the proteome of FSHD myotubes that are predominantly atrophic or disorganized, and could detect clear differences in their protein relative abundance profiles. It could be expected that primary cultures containing more similar proportions of atrophic and disorganized myotubes will exhibit an intermediate proteomic profile, as observed in analysis #P1.

Atrophic myotubes display a general decrease in the relative abundance of cytoskeletal and contractile proteins that could reflect atrophy-associated proteolysis and differentiation defects. We have previously used the same cellular model [30] and have found that DUX4 is expressed in a higher number of myonuclei in FSHD myotubes than in control myotubes and that the level of DUX4 expression is higher in the myonuclei of atrophic myotubes. As we have described in [20], the induction of E3 ubiquitin ligases MURF1 and atrogin-1/MAFBX by DUX4 may cause muscle atrophy in FSHD. These data were recently confirmed and expanded by the identification of DUX4 direct target genes in human myoblasts using a combined transcriptome and chromatin immunoprecipitation study [19]. Consistent with our findings, these researchers have found that multiple ubiquitin ligase family members are induced by DUX4. In addition, DUX4 induces DEFB103, which is a human defensin that up-regulates myostatin and inhibits muscle differentiation. Muscle cultures exposed to DEFB103 showed decreased expression of myosin heavy chains [19]. In the present study, we also observed a clear lower relative abundance of most myosin heavy and light chain isoforms that are typical of skeletal muscle. Because myosin is a marker of terminal myogenic differentiation, its downregulation in FSHD myotubes is in agreement with a dampening of normal myogenesis, as described in [26]. Accordingly, as described in [31], we observed an increased level of vimentin in FSHD myotubes, which is an intermediate filament component that is normally downregulated during myogenic differentiation.

Our data also suggest a myosin isoform switch in FSHD atrophic myotubes in favor of non-muscle myosin complexes. Those myosin isoforms are differentially expressed, and their isoform-specific expression is cell type-dependent and linked to cell proliferation and differentiation [40]. Because of their position downstream of convergent signaling pathways, NMHCs play a central role in the control of cell adhesion, cell migration and tissue architecture [41], and they are involved in secretion, membrane trafficking and repair [42]. NMHCs also drive changes in cell morphology during myoblast alignment and fusion [43]. The upregulation of non-muscle myosin in FSHD, together with the upregulation of thrombospondin, GPI anchor Thy-1 glycoprotein, filamin-A, vimentin and perlecan, suggests that cell adhesion is altered in FSHD myotubes. Taken together with the perturbations associated with the actin cytoskeleton and caveolar proteins, the present data support the hypothesis of a trafficking and membrane repair dysfunction in FSHD.

### Disorganized Myotubes Appear to Resist DUX4-mediated Damage

Our results are in agreement with an induction of the atrophic phenotype by DUX4, but the molecular mechanism that underlies the disorganized phenotype appears to be more complex. In contrast to atrophic myotubes, the categories of dysregulated proteins are more heterogeneous and the decrease in protein relative abundance is more moderate. Disorganized myotubes express DUX4 in a similar number of myonuclei when compared with atrophic myotubes. However, the number of nuclei with intense DUX4 staining is significantly lower [30], which could partially explain why the contractile muscle component is not reduced to the extent that it is reduced in the atrophic phenotype. Rather than exhibiting a proteolytic mechanism that occurs in an atrophic process, cytoskeletal proteins appear to have a disrupted spatial organization (data not shown). Accordingly, we observed increased levels of proteins linked to cytoskeleton organization, particularly those that are involved in the re-arrangement of microtubule network components such as tubulin, αß-crystallin and heat shock proteins. Indeed, these proteins are known to interact and contribute to dynamic microtubule reorganization during adaptation phases in skeletal muscle [44]. The relative expression of αß-crystallin and other heat shock proteins is also increased early after intense exercise as a remodeling response to limit the extent of muscle damage that is incurred upon mechanical stress [45]. In FSHD, as opposed to a downregulation as observed in a rat model of muscle atrophy [44], we suggest that the upregulation of those proteins in FSHD disorganized myotubes could constitute a compensatory mechanism against DUX4-mediated atrophy. Intriguingly, a similar phenotype of giant myotubes with myonuclei clusters was observed after cholesterol depletion [46]. The microtubule distribution was maintained in this model, where the disorganized myotube morphology was attributed to an enhanced myogenic fusion that was linked to an alteration of membrane microdomains enriched in cholesterol such as caveolae. Similarly, our study highlights perturbations in the relative abundance of several caveolar proteins in FSHD myotubes. A defect in these membrane microdomain subtypes could also contribute to myotube deformation in FSHD.

Because the alteration of caveolar proteins was also found in atrophic myotubes, further studies are necessary to precisely determine the contribution of each factor to the formation of a given phenotype. Because the predominantly atrophic or disorganized FSHD cultures that we have analyzed are derived from comparable patients in terms of the number of *D4Z4* units, sex and age, we assume that other factors could intervene to explain the emergence of a non-atrophic phenotype, despite the expression of DUX4. Other genes were suggested to be involved in FSHD, including *FRG1* [8], *ANT1* [7] and *DUX4c* [10], but further studies are necessary to explain the relative contribution of each *4q35* gene in FSHD [19]. DUX4c is induced in FSHD muscles [10] and could bind to DUX4-target promoters through its identical double homeodomain, as was described for PITX1 [12]. Because DUX4c overexpression is associated with increased myoblast proliferation and decreased differentiation, it is a good candidate to explain the emergence of a non-atrophic phenotype. DUX4-s, which is a putative protein derived from a short *DUX4* mRNA variant that is often detected in control muscles and less frequently in FSHD muscles [15], was suggested to act as a dominant negative variant [19]. DUX4-s may also take part in this process, but further studies are needed to determine whether this protein is endogenously expressed in FSHD or control muscle cells.

### FSHD Pathogenesis Likely Involves Perturbations of Membrane Microdomains

The present data indicate that FSHD myotubes present clear changes in the relative abundance of proteins typical of caveolae (i.e., PTRF, MURC, and Gpi-anchor proteins such as THY1), which are membrane lipid microdomains that are enriched in cholesterol and glycosphingolipids and are often considered to be a specialized lipid raft subtype. These membrane invaginations play a major role in signal transduction and appear to constitute signaling platforms that mediate the sequestration of certain receptors, transporters and signaling proteins [47]. They are thus involved in numerous biological processes, e.g., membrane repair, redox signaling, immune response and lipid metabolism. Caveolin-3 (CAV3) is the main caveolar protein in skeletal muscle and is a key factor in muscle cell fusion, and several mutations in the CAV3 gene cause heterogeneous neuromuscular diseases including caveolinopathies such as LGMD1 (limb girdle muscular dystrophy) [47], [48]. Caveolin-associated cavins, particularly PTRF/cavin-1, are crucial regulators of caveola formation [49]. MURC/cavin-4 was first described as a cytosolic protein that is partly localized in the Z-line and associated with cardiac dysfunction through the modulation of the Rho/ROCK pathway [50]. MURC expression is increased during the differentiation of C2C12 myoblasts, and its RNAi-mediated knockdown impairs myogenic differentiation [39]. In the present study, we reported a decreased level of MURC in FSHD myotubes. FSHD myoblasts fail to upregulate MURC during their differentiation, and this perturbation could also be linked to the general dampening of myogenic differentiation associated with FSHD as described in [26]. Recently, MURC was found to be localized to sarcolemmal caveolae in normal muscle, with an impaired distribution in muscle from a patient with heterogeneous CAV3 expression [51], which suggests a potential role of MURC in caveolin-associated muscle disease.

In conclusion, the use of an optimized proteomic approach has enabled us to define molecular differences between atrophic and disorganized FSHD myotubes. Observed changes are likely consequences of dysregulation cascades initiated by misexpression of *4q35* genes. Atrophic myotubes presented molecular characteristics that are typically observed following DUX4 expression. Conversely, disorganized myotubes presented increased levels of proteins involved in microtubule network organization and myofibrillar remodeling, which suggests a compensatory response to DUX4-mediated damage. Further studies are necessary to determine the relative contribution of other *4q35* genes leading to this phenotype. Moreover, our results suggest that FSHD pathogenesis could partially involve a defect of membrane microdomains as observed in other neuromuscular disorders. Finally, the study of a fraction enriched in nuclear proteins suggested a defect in RNA processing in FSHD myotubes.

## Materials and Methods

### Ethics Statement

Primary human myoblasts were obtained according to procedures approved by the current ethical and legislative rules of France or Belgium, and written informed consent was obtained from all subjects, as directed by the ethical committee of CHU de Villeneuve (Montpellier, France) [27]. In addition, the use of this material has been approved by the ethics committee of the University of Mons (ref# A901). To assess whether the biopsied muscle was affected and to evaluate the severity of the pathology, we used clinical and histopathologic criteria as described elsewhere [27].

### Cell Culture

Primary myoblast cultures from control individuals and patients with FSHD were isolated from muscle biopsies, purified by selection of CD56^+^ cells and established as described in [27]. They were grown in collagen-coated dishes (Iwaki, Tokyo, Japan) in DMEM with high glucose (4.5 g/l), sodium pyruvate and sodium bicarbonate (Sigma-Aldrich, St. Louis, MO, USA) with L-glutamine (4 mM, Sigma-Aldrich), gentamycin (50 µg/ml, Sigma-Aldrich), 10% fetal bovine serum (FBS, Invitrogen), and 1% Ultroser G (Pall BioSepra, Cergy-St-Christophe, France) at 37°C under 5% CO_2_. Before experimentation, primary myoblasts were seeded in 10-cm or 35-mm collagen-coated dishes for Western blot or immunofluorescence, respectively, in DMEM/gentamycin (50 µg/ml)/FBS (20%). Myogenic differentiation of confluent cells was induced by decreasing the FBS concentration to 2%.

### Protein Extraction

Total extracts and a fraction enriched in nuclear proteins were both analyzed following the procedure described in the workflow scheme (**Fig. 1**).

For total protein extraction (TE), cells were washed 3 times in cold PBS. Cellular pellets obtained by centrifugation were then stored at −80°C.

For nuclear extracts (NE), after the excess PBS was removed, the cytoplasmic extract was prepared directly on the cell dish using the NE-PER Nuclear and Cytoplasmic Extraction Reagent kit (Thermo Scientific, Rockford, IL, USA) at 4°C according to the manufacturer’s instructions. The resulting nuclear pellet was resuspended and subjected to ultracentrifugation (30,000 g for 45 min at 4°C) on a sucrose cushion (1.8 M), followed by 2 washes at 4°C. The nuclear pellet was then stored at −80°C.

Cellular and nuclear pellets were then dissolved in 6 M guanidinium chloride (lysis buffer of the ICPL kit, SERVA, Germany), followed by ultra-sonication for 3x15 s (80% amplitude, U50 IKAtechnik) and incubation for 20 min at room temperature (RT). The supernatant was harvested after centrifugation (18,000 g for 15 min at RT), and the protein concentration was determined according to the Bradford method using bovine gamma-globulin as a standard.

### ICPL Labeling

For the ICPL procedure, 100 µg of protein was labeled using the ICPL kit (SERVA) following the manufacturer’s instructions as described in [52]. Briefly, after reduction and alkylation, proteins were labeled at the protein NH_2_-termini and lysine side chains by incubation for 2 h at RT with a light (L, control samples) or heavy (H, FSHD samples) form of the ICPL reactant (nicotinoyl-N-hydroxysuccinimide). After the excess reactant was quenched with hydroxylamine, the control and FSHD samples were pooled. The proteins were recovered through acetone precipitation and dissolved in 50 mM Tris-HCl, pH 7.5, with 2 M urea. The proteins were then digested using trypsin at an enzyme/substrate ratio of 1∶50 for 4 h at 37°C.

The post-digest ICPL procedure was performed as described in [52]. 33 µg of protein was reduced; alkylated; recovered by acetone precipitation; dissolved in 100 mM phosphate buffer, pH 8.5, with 1 M urea and subjected to trypsin digestion for 5 h at 37°C (enzyme:substrate ratio 1∶25). Tryptic peptides were then labeled with the ICPL reactant for 3 h according to [52]. The reaction was stopped, and the samples were mixed as described above.

### LC-MS/MS Analysis

24 µg of tryptic peptides was subjected to online 2D-LC MS/MS analysis as described in [52], with SCX as the first dimension of separation (analysis #P1: SCX, POROS10S, 10 cm, Dionex, The Netherlands; analysis #P2, #P3, aFSHD_TE/NE and dFSHD12_TE/NE: Biobasic SCX, Thermo) and RP column (75 µm ID x 15 cm PepMap C18 column, Dionex) as the second dimension of separation. Peptides were sequentially eluted from SCX with plugs of increasing NaCl concentration (1, 2.5, 5, 10, 25, 50, 100, 200, and 1000 mM) and NH_4_Cl concentration (5, 10, 25, 50, 75, 100, 125, 150, 200, 400 and 800 mM) in the loading solvent for ICPL and post-digest ICPL, respectively. Eluted peptides were separated on the RP column using an acetonitrile gradient of 5–35% for 120 min. MS data were acquired on a HCT ultra ion trap mass spectrometer (Bruker, Germany) as previously described [52]. ICPL data were processed using Bruker Daltonics software Data analysis 2.4 with default parameters, whereas post-digest ICPL data were processed using MASCOT distiller 2.3.2. The created peak list was used as the input for Mascot MS/MS ion searches using an in-house Mascot 2.2 server (Matrix Science) against a human-restricted SwissProt database. The search parameters were enzyme: trypsin; max. missed cleavages  = 2; fixed modifications = carbamidomethyl (C); variable modifications = oxidation (M); ICPL modification at both peptide N-ter and lysine side chains; peptide tolerance ±1.3 Da; MS/MS tolerance ±0.5 Da; peptide charge  = 2+ and 3+; and instrument = ESI-TRAP. Only proteins identified with a protein score above the calculated Mascot ion score, which was defined at the 95% confidence level, were considered.

The MASCOT distiller was used for protein quantification with the following parameters: integration method: simple; correlation threshold: 0.8; standard error threshold: 999; XIC threshold: 0.2; max XIC width: 7; fraction threshold: 0.5 and mass time matches allowed. Only peptides with an ion score above 30 were considered for quantification. Quantitative data were systematically inspected manually, and outlier ratios were manually recalculated. Protein ratios for which coefficients of variation were greater than 25% or quantified based on less than 3 peptides were also manually recalculated as described in [52].

The dispersion value for proteins represented by multiple peptides is represented by the geometric standard deviation (SD). A non-normal distribution is noted as NN in the SD column of the tables. The false-positive rates, determined using a Decoy database as described [53] are as follows: 0.65% (dFSHD12_NE), 0.77% (dFSHD12_TE), 0.13% (aFSHD3_NE) and 0.81% (aFSHD3_TE). Proteins changes were considered significant when the fold change was greater than 1.5 or less than 0.7 as described in [54], [55]. At the individual level, fold changes were assessed using Student’s *t*-test (*t*-test column in tables: *). For classification into functional categories, proteins with fold changes <0.8 or >1.3 were analyzed using bioinformatic tools, including the Database for Annotation, Visualization and Integration (DAVID), Ingenuity Pathway Analysis (IPA), UniProtKB. In DAVID analyses, the p-value is equivalent to the EASE score, which uses a conservative adjustment of the Fisher’s exact probability, and was applied to identify significantly enriched gene categories (*: p<0.05).

### Western Blot Immunodetection

Whole-cell extracts of myoblast primary cultures were obtained by lysis in hypertonic buffer (50 mM Tris, pH 7.0, 50 mM NaCl, 0.1% Nonidet P40, 1 mM DTT and protease inhibitor cocktail (Sigma-Aldrich)) and 3 freeze/thaw cycles. The cell lysate was separated by 12% SDS-PAGE for 3 to 4 h at 100V and electrotransferred onto a nitrocellulose membrane (GE Healthcare Europe GmbH, Diegem, Belgium). The membrane was stained with Ponceau red to check loading and migration quality, and an image was captured for the loading control. After being rinsed in PBS, the membrane was blocked for 1 h at room temperature (RT) in phosphate-buffered saline (PBS) with 5% non-fat dry milk, rinsed in PBS and incubated overnight at 4°C with primary antibodies. The following antibodies and dilutions were used: rabbit polyclonal anti-PABP4 (Bethyl A301-466A, 1∶2000 in PBS-5% milk), rabbit polyclonal anti-MURC (Sigma-Aldrich HPA021021, 1∶2000 in PBS-5% milk), rabbit polyclonal anti-PTRF (Bethyl A301-269A, 1∶2000 in PBS-5% milk). After rinsing in PBS, appropriate secondary antibodies coupled to HRP (1∶5000, GE Healthcare) were added and detected with the Super Signal West Femto Maximum Sensitivity Substrate (Pierce) or Lumilight (Roche Diagnostics) on Amersham Hyperfilm ECL (GE Healthcare). For standardization, membranes were stripped, and immunoreactive bands were visualized with an anti-GAPDH MAb (Applied Biosystems Ambion, 1∶4000 in PBS-2% BSA). Densitometry of the immunoreactive bands was performed using labImage ID Software (Kapelan Bio-Imaging). Data are normalized to control loading levels for each sample. Antibody specificity was evaluated by competition using pre-incubation of the antibody with a 5-fold excess of blocking peptides for PTRF (Bethyl BP301-269, 1∶400) and PABP4 (Bethyl BP301-466, 1∶400).

### Immunofluorescence

Primary myoblasts seeded on 35-mm collagen-coated dishes (Iwaki, Japan) were fixed for 5 min at room temperature (RT) in 4% paraformaldehyde. Cell permeabilization was performed in PBS-0.5% Triton X-100 for 5 min at RT. After blocking in PBS-20% FBS, cells were incubated with primary antibodies for 2 h at RT. The following antibodies and dilutions were used: rabbit polyclonal anti-PABP4 (Bethyl A301-466A, 1∶100 in PBS), rabbit polyclonal anti-MURC (Sigma-Aldrich HPA021021, 1∶100 in PBS), rabbit polyclonal anti-PTRF (Bethyl A301-269A, 1∶100 in PBS), MAb 9A12 (hybridoma supernatant: 1∶1), and anti-caveolin3 MAb (BD Biosciences 610420, 1∶100 in PBS). After washing and blocking, cells were incubated for 1 h at RT with the Alexa Fluor secondary antibodies goat anti-mouse 488 and anti-rabbit 555 (1/100, Invitrogen). After washing, coverslips were applied over antifade reagent with 4,6-diamidino-2-phenylindole (DAPI, Invitrogen). The PTRF antibody specificity was confirmed by peptide competition (data not shown).

Microscopy images were acquired using a Nikon Microscope Eclipse 80i with a DS-U3 DS camera control unit and the NIS element-BR analysis software. Plan Fluor 20x and Plan Fluor 40x and objectives were used with 350-, 480- and 540-nm excitation for the 4,6-diamidino-2-phenylindole (DAPI), fluorescein isothiocyanate (FITC), and tetramethylrhodamine isothiocyanate (TRITC) channels, respectively.

## Supporting Information

Figure S1 Comparison of protein composition in total (TE) and nuclear protein-enriched (NE) fractions of FSHD myotubes (analysis #P2 and P3). The analysis was conducted using TE and NE of FSHD myotubes (dFSHD12) 4 days after differentiation by 2DLC-MS/MS without ICPL labeling. **(A)** Subcellular classification of the detected proteins in TE and NE of FSHD myotubes. **(B)** Histograms comparing the number of detected proteins in TE and NE of FSHD myotubes and their subcellular localizations. **(C)** Functional classification of nuclear proteins detected in FSHD myotubes. The subcellular and functional classifications were conducted using ingenuity pathway analysis (IPA) or David database bioinformatics tools, respectively.
(TIF)

Figure S2 The polyA-binding protein 1/4 (PABP1/4) is up-regulated in FSHD myotubes **(A)** MS spectrum of the AHLTNQYMQ peptide that is common to PABP1 and PABP4 proteins. The graph represents the isotopic distribution corresponding to the FSHD peptide labeled with the heavy ICPL tag (right) and the control peptide labeled with the light ICPL tag (left). The H/L intensity ratio of 18.74 corresponds to the relative protein quantification. The theoretical and experimental spectra are indicated in red or black, respectively. **(B)** Western blot analysis of TE of atrophic (aFSHD3), disorganized (dFSHD12) and control (CTL12) myotubes using an antibody directed against PABP4 (Bethyl Laboratories). The bottom panel corresponds to the densitometry analysis. **(C)** Specificity of the anti-PABP4 antibody. Immortalized human myoblasts were kindly provided by Drs. G. Butler-Browne and V. Mouly (Institute of Myology, Paris). These lines were derived from a non-affected control (LHCN-M2) and were immortalized as described in [56]. They were cultivated and differentiated for 4 days, as described in [20]. Putative regulation by proteolytic degradation was evaluated by adding the proteasome inhibitor MG132 (25 µM, Sigma Aldrich) to the culture medium 5 h before the cells were harvested. Total cell protein extracts (20 µg, RIPA buffer) was separated by 12% SDS-PAGE, transferred to a nitrocellulose membrane and immunodetected with the anti-PABP4 antibody. A band at the expected MW for PAPB4 was detected, and this signal disappeared upon competition with a 5-fold excess of the antigenic peptide (+Ag, Bethyl Laboratories). The addition of MG132 slightly improved PABP4 detection.
(TIF)

Figure S3 Changes in the 14-3-3 protein epsilon (YWHAE) intracellular distribution suggest a disruption of its nuclear-cytoplasmic shuttling in FSHD myotubes. Representative MS spectrum of the 14-3-3 protein epsilon peptide quantified by 2DLC-MS/MS in TE and NE of aFSHD3 myotubes. The graph represents the isotopic distribution corresponding to the FSHD peptide labeled with the heavy ICPL tag (right) and the control peptide labeled with the light ICPL tag (left). The indicated H/L intensity ratios correspond to the relative protein quantification.
(TIF)

Figure S4 Quantification of the serine protease HTRA1 suggests the presence of two isoforms, and only one appears to have increased expression in atrophic FSHD myotubes. Representative MS spectra of HTRA1 peptides quantified by 2DLC-MS/MS in TE of aFSHD3 myotubes. The graph represents the isotopic distribution corresponding to the FSHD peptide labeled with the heavy ICPL tag (right) and the control peptide labeled with the light ICPL tag (left). The indicated H/L intensity ratios correspond to the relative protein quantification.
(TIF)

Table S1 Summary of published transcriptomic and proteomic studies on FSHD myoblasts and muscle biopsies. d5-7: 5 to 7 days of differentiation; d8: 8 days of differentiation.
(DOCX)

Table S2 Patient characteristics and 2DLC-MS/MS analysis. **(A)** Name of the FSHD cell line (code) as indicated in [27] (“line” refers to a myoblast population derived from a single biopsy; a: predominantly atrophic myotubes; d: predominantly disorganized myotubes); age and sex of the patient (M: male; F: female); number of D4Z4 units; site of the muscle biopsy [Q = quadriceps (vastus lateralis)]; score on the Brooke–Vignos scale defining the clinical status of upper and lower limb muscles, respectively, where high values define affected muscles and low values define non-affected muscles; predominant phenotype of the derived myotubes and MFI determined in [27] (myoblast fusion index: ratio between the nuclei present in myotubes versus the total number of nuclei in a given microscope field; the proportion of atrophied myotubes in a culture is inversely correlated with the MFI). **(B)** The following information is indicated for each 2DLC-MS/MS analysis: the FSHD and control myoblasts line that was compared, the differentiation stage (d4: 4 days; d6: 6 days), the extraction type (TE: total extracts; NE: fraction enriched in nuclear proteins), the ICPL procedure (regular or Post-digest), the SCX column (P: POROS10S, Dionex; B: Biobasic SCX, Thermo), the number of identified and quantified proteins and the total number of non-redundant identified peptides.
(DOCX)

Table S3 Quantitative proteomics data for dFSHD12_TE/NE and aFSHD3_TE/NE analyses. Quantitative data are given for proteins for which an H/L ratio greater than 1.3 or lower than 0.8 was observed. AC: UniProt accession number; Hugo Gene symbol; Protein name; H/L: fold change; SD: geometric standard deviation; #: number of peptides used for quantification; T. Test: statistical significance assessed by Student’s *t*-test (*: p<0.05). These proteins were classified using the DAVID database (functional annotation charts, GOTERM_BP_FAT) and categories with a significant p-value (Ease Score) are indicated in the last column. The proteins with a fold change >1.5 are highlighted in red, >1.2 in pink, <0.7 in green, and 0.7–0.8 in light green. TE: total extract; NE: nuclear extract.
(XLSX)

Table S4 Quantitative proteomics data (A) and bibliographic information related to myosin heavy (B) and light (C) chain isoforms. **(A)** AC: UniProt accession number; Score: -10*Log (P), where P is the probability that the observed match is a random event; H/L: fold change; SD: geometric standard deviation; #: number of peptides used for quantification; *: statistical significance assessed by Student’s *t*-test (p<0.05). The proteins with a fold change >1.5 are highlighted in red, >1.2 in pink, <0.7 in green, and 0.7–0.8 in light green. TE: total extract; NE: fraction enriched in nuclear proteins. **(B)** Information related to myosin heavy chain (MYH: upper panel) types, functions and mutations as given in the UniProt database and in [57]. The role of the non-muscle myosin MYH9 in myoblast differentiation was described in [58]. **(C)** Biological processes that involve myosin light chains (MYL: bottom panel) were identified in the UniProt database (Gene Ontology annotation).
(XLSX)

Table S5 Raw data: analysis #P1.
(XLSX)

Table S6 Raw data: analysis #P2 and #P3.
(XLSX)

Table S7 Raw data: analysis #aFSHD3_TE.
(XLSX)

Table S8 Raw data: analysis #aFSHD3_NE.
(XLSX)

Table S9 Raw data: analysis #dFSHD12_TE.
(XLSX)

Table S10 Raw data: analysis #dFSHD12_NE.
(XLSX)

Table S11 Raw data: analysis #CTL7_TE.
(XLSX)

Text S1 Regular ICPL coupled to 2DLC-MS/MS (Analysis #P1).
(DOCX)

Text S2 Myotube nuclear enrichment to improve protein detection (Analysis #P2-3).
(DOCX)

Text S3 Post-digest ICPL to improve protein quantification in FSHD myotubes.
(DOCX)

Text S4 Validation of the increased amount of PABP in FSHD myotubes.
(DOCX)

## References

[1] Hewitt JE, Lyle R, Clark LN, Valleley EM, Wright TJ, et al (1994) Analysis of the tandem repeat locus D4Z4 associated with facioscapulohumeral muscular dystrophy. Hum Mol Genet 3: 1287–1295. 7987304 10.1093/hmg/3.8.1287

[2] Wijmenga C, Hewitt JE, Sandkuijl LA, Clark LN, Wright TJ, et al (1992) Chromosome 4q DNA rearrangements associated with facioscapulohumeral muscular dystrophy. Nat Genet 2: 26–30 doi:10.1038/ng0992-26 1363881 10.1038/ng0992-26

[3] Richards M, Coppée F, Thomas N, Belayew A, Upadhyaya M (2011) Facioscapulohumeral muscular dystrophy (FSHD): an enigma unravelled? Hum Genet. Available:http://www.ncbi.nlm.nih.gov/pubmed/21984394. Accessed 19 December 2011. 10.1007/s00439-011-1100-z 21984394

[4] van Overveld PGM, Lemmers RJFL, Sandkuijl LA, Enthoven L, Winokur ST, et al (2003) Hypomethylation of D4Z4 in 4q-linked and non-4q-linked facioscapulohumeral muscular dystrophy. Nat Genet 35: 315–317 doi:10.1038/ng1262 14634647 10.1038/ng1262

[5] Petrov A, Pirozhkova I, Carnac G, Laoudj D, Lipinski M, et al (2006) Chromatin loop domain organization within the 4q35 locus in facioscapulohumeral dystrophy patients versus normal human myoblasts. Proc Natl Acad Sci USA 103: 6982–6987 doi:10.1073/pnas.0511235103 16632607 10.1073/pnas.0511235103 PMC1459005

[6] Cabianca DS, Casa V, Bodega B, Xynos A, Ginelli E, et al (2012) A Long ncRNA Links Copy Number Variation to a Polycomb/Trithorax Epigenetic Switch in FSHD Muscular Dystrophy. Cell 149: 819–831 . 22541069 10.1016/j.cell.2012.03.035 PMC3350859

[7] Laoudj-Chenivesse D, Carnac G, Bisbal C, Hugon G, Bouillot S, et al (2005) Increased levels of adenine nucleotide translocator 1 protein and response to oxidative stress are early events in facioscapulohumeral muscular dystrophy muscle. J Mol Med 83: 216–224 doi:10.1007/s00109-004-0583-7 15551024 10.1007/s00109-004-0583-7

[8] Gabellini D, D’Antona G, Moggio M, Prelle A, Zecca C, et al (2006) Facioscapulohumeral muscular dystrophy in mice overexpressing FRG1. Nature 439: 973–977 doi:10.1038/nature04422 16341202 10.1038/nature04422

[9] Rijkers T, Deidda G, van Koningsbruggen S, van Geel M, Lemmers RJLF, et al (2004) FRG2, an FSHD candidate gene, is transcriptionally upregulated in differentiating primary myoblast cultures of FSHD patients. J Med Genet 41: 826–836 doi:10.1136/jmg.2004.019364 15520407 10.1136/jmg.2004.019364 PMC1735617

[10] Ansseau E, Laoudj-Chenivesse D, Marcowycz A, Tassin A, Vanderplanck C, et al (2009) DUX4c is up-regulated in FSHD. It induces the MYF5 protein and human myoblast proliferation. PLoS ONE 4: e7482 doi:10.1371/journal.pone.0007482 19829708 10.1371/journal.pone.0007482 PMC2759506

[11] Gabriëls J, Beckers MC, Ding H, De Vriese A, Plaisance S, et al (1999) Nucleotide sequence of the partially deleted D4Z4 locus in a patient with FSHD identifies a putative gene within each 3.3 kb element. Gene 236: 25–32. 10433963 10.1016/s0378-1119(99)00267-x

[12] Dixit M, Ansseau E, Tassin A, Winokur S, Shi R, et al (2007) DUX4, a candidate gene of facioscapulohumeral muscular dystrophy, encodes a transcriptional activator of PITX1. Proc Natl Acad Sci USA 104: 18157–18162 doi:10.1073/pnas.0708659104 17984056 10.1073/pnas.0708659104 PMC2084313

[13] Lemmers RJLF, van der Vliet PJ, Klooster R, Sacconi S, Camaño P, et al (2010) A unifying genetic model for facioscapulohumeral muscular dystrophy. Science 329: 1650–1653 doi:10.1126/science.1189044 20724583 10.1126/science.1189044 PMC4677822

[14] Snider L, Asawachaicharn A, Tyler AE, Geng LN, Petek LM, et al (2009) RNA transcripts, miRNA-sized fragments and proteins produced from D4Z4 units: new candidates for the pathophysiology of facioscapulohumeral dystrophy. Hum Mol Genet 18: 2414–2430 doi:10.1093/hmg/ddp180 19359275 10.1093/hmg/ddp180 PMC2694690

[15] Snider L, Geng LN, Lemmers RJLF, Kyba M, Ware CB, et al (2010) Facioscapulohumeral dystrophy: incomplete suppression of a retrotransposed gene. PLoS Genet 6: e1001181 doi:10.1371/journal.pgen.1001181 21060811 10.1371/journal.pgen.1001181 PMC2965761

[16] Kowaljow V, Marcowycz A, Ansseau E, Conde CB, Sauvage S, et al (2007) The DUX4 gene at the FSHD1A locus encodes a pro-apoptotic protein. Neuromuscul Disord 17: 611–623 doi:10.1016/j.nmd.2007.04.002 17588759 10.1016/j.nmd.2007.04.002

[17] Bosnakovski D, Xu Z, Gang EJ, Galindo CL, Liu M, et al (2008) An isogenetic myoblast expression screen identifies DUX4-mediated FSHD-associated molecular pathologies. EMBO J 27: 2766–2779 doi:10.1038/emboj.2008.201 18833193 10.1038/emboj.2008.201 PMC2572182

[18] Wallace LM, Garwick SE, Mei W, Belayew A, Coppee F, et al (2011) DUX4, a candidate gene for facioscapulohumeral muscular dystrophy, causes p53-dependent myopathy in vivo. Ann Neurol 69: 540–552 doi:10.1002/ana.22275 21446026 10.1002/ana.22275 PMC4098764

[19] Geng LN, Yao Z, Snider L, Fong AP, Cech JN, et al (2012) DUX4 activates germline genes, retroelements, and immune mediators: implications for facioscapulohumeral dystrophy. Dev Cell 22: 38–51 doi:10.1016/j.devcel.2011.11.013 22209328 10.1016/j.devcel.2011.11.013 PMC3264808

[20] Vanderplanck C, Ansseau E, Charron S, Stricwant N, Tassin A, et al (2011) The FSHD Atrophic Myotube Phenotype Is Caused by DUX4 Expression. PLoS ONE 6: e26820 doi:10.1371/journal.pone.0026820 22053214 10.1371/journal.pone.0026820 PMC3203905

[21] Tupler R, Perini G, Pellegrino MA, Green MR (1999) Profound misregulation of muscle-specific gene expression in facioscapulohumeral muscular dystrophy. Proc Natl Acad Sci USA 96: 12650–12654. 10535977 10.1073/pnas.96.22.12650 PMC23032

[22] Winokur ST, Chen Y-W, Masny PS, Martin JH, Ehmsen JT, et al (2003) Expression profiling of FSHD muscle supports a defect in specific stages of myogenic differentiation. Hum Mol Genet 12: 2895–2907 doi:10.1093/hmg/ddg327 14519683 10.1093/hmg/ddg327

[23] Osborne RJ, Welle S, Venance SL, Thornton CA, Tawil R (2007) Expression profile of FSHD supports a link between retinal vasculopathy and muscular dystrophy. Neurology 68: 569–577 doi:10.1212/01.wnl.0000251269.31442.d9 17151338 10.1212/01.wnl.0000251269.31442.d9

[24] Arashiro P, Eisenberg I, Kho AT, Cerqueira AMP, Canovas M, et al (2009) Transcriptional regulation differs in affected facioscapulohumeral muscular dystrophy patients compared to asymptomatic related carriers. Proc Natl Acad Sci USA 106: 6220–6225 doi:10.1073/pnas.0901573106 19339494 10.1073/pnas.0901573106 PMC2664154

[25] Celegato B, Capitanio D, Pescatori M, Romualdi C, Pacchioni B, et al (2006) Parallel protein and transcript profiles of FSHD patient muscles correlate to the D4Z4 arrangement and reveal a common impairment of slow to fast fibre differentiation and a general deregulation of MyoD-dependent genes. Proteomics 6: 5303–5321 doi:10.1002/pmic.200600056 17013991 10.1002/pmic.200600056

[26] Tsumagari K, Chang S-C, Lacey M, Baribault C, Chittur SV, et al (2011) Gene expression during normal and FSHD myogenesis. BMC Med Genomics 4: 67 doi:10.1186/1755-8794-4-67 21951698 10.1186/1755-8794-4-67 PMC3204225

[27] Barro M, Carnac G, Flavier S, Mercier J, Vassetzky Y, et al (2010) Myoblasts from affected and non-affected FSHD muscles exhibit morphological differentiation defects. J Cell Mol Med 14: 275–289 doi:10.1111/j.1582-4934.2008.00368.x 18505476 10.1111/j.1582-4934.2008.00368.x PMC2910739

[28] Cheli S, François S, Bodega B, Ferrari F, Tenedini E, et al (2011) Expression profiling of FSHD-1 and FSHD-2 cells during myogenic differentiation evidences common and distinctive gene dysregulation patterns. PLoS ONE 6: e20966 doi:10.1371/journal.pone.0020966 21695143 10.1371/journal.pone.0020966 PMC3113851

[29] Eisenberg I, Eran A, Nishino I, Moggio M, Lamperti C, et al (2007) Distinctive patterns of microRNA expression in primary muscular disorders. Proc Natl Acad Sci USA 104: 17016–17021 doi:10.1073/pnas.0708115104 17942673 10.1073/pnas.0708115104 PMC2040449

[30] Tassin A, Laoudj-Chenivesse D, Vanderplanck C, Barro M, Charron S, et al.. (2012) DUX4 expression in FSHD muscle cells: how could such a rare protein cause a myopathy? J Cell Mol Med. In press. 10.1111/j.1582-4934.2012.01647.x PMC3823138 23206257

[31] Dmitriev PV, Barat AL, Cochet E, Ogryzko VV, Laoudj-Chenivesse D, et al. (2011) FSHD myoblasts fail to downregulate intermediate filament protein vimentin during myogenic differentiation. Biopolymers and Cell. Available:http://www.doaj.org/doaj?func=abstract&id=867355. Accessed 2012 Mar 25.

[32] Dzijak R, Yildirim S, Kahle M, Novák P, Hnilicová J, et al (2012) Specific nuclear localizing sequence directs two myosin isoforms to the cell nucleus in calmodulin-sensitive manner. PLoS ONE 7: e30529 doi:10.1371/journal.pone.0030529 22295092 10.1371/journal.pone.0030529 PMC3266300

[33] Chen M, Shen X (2007) Nuclear actin and actin-related proteins in chromatin dynamics. Curr Opin Cell Biol 19: 326–330 doi:10.1016/j.ceb.2007.04.009 17467255 10.1016/j.ceb.2007.04.009

[34] Ketema M, Wilhelmsen K, Kuikman I, Janssen H, Hodzic D, et al (2007) Requirements for the localization of nesprin-3 at the nuclear envelope and its interaction with plectin. J Cell Sci 120: 3384–3394 doi:10.1242/jcs.014191 17881500 10.1242/jcs.014191

[35] Rando OJ, Zhao K, Crabtree GR (2000) Searching for a function for nuclear actin. Trends Cell Biol 10: 92–97. 10675902 10.1016/s0962-8924(99)01713-4

[36] Chien J, Aletti G, Baldi A, Catalano V, Muretto P, et al (2006) Serine protease HtrA1 modulates chemotherapy-induced cytotoxicity. J Clin Invest 116: 1994–2004 doi:10.1172/JCI27698 16767218 10.1172/JCI27698 PMC1474818

[37] Park I, Han C, Jin S, Lee B, Choi H, et al (2011) Myosin regulatory light chains are required to maintain the stability of myosin II and cellular integrity. Biochem J 434: 171–180 doi:10.1042/BJ20101473 21126233 10.1042/BJ20101473

[38] Briand N, Dugail I, Le Lay S (2011) Cavin proteins: New players in the caveolae field. Biochimie 93: 71–77 doi:10.1016/j.biochi.2010.03.022 20363285 10.1016/j.biochi.2010.03.022

[39] Tagawa M, Ueyama T, Ogata T, Takehara N, Nakajima N, et al (2008) MURC, a muscle-restricted coiled-coil protein, is involved in the regulation of skeletal myogenesis. Am J Physiol, Cell Physiol 295: C490–498 doi:10.1152/ajpcell.00188.2008 18508909 10.1152/ajpcell.00188.2008

[40] Golomb E, Ma X, Jana SS, Preston YA, Kawamoto S, et al (2004) Identification and characterization of nonmuscle myosin II-C, a new member of the myosin II family. J Biol Chem 279: 2800–2808 doi:10.1074/jbc.M309981200 14594953 10.1074/jbc.M309981200

[41] Vicente-Manzanares M, Ma X, Adelstein RS, Horwitz AR (2009) Non-muscle myosin II takes centre stage in cell adhesion and migration. Nat Rev Mol Cell Biol 10: 778–790 doi:10.1038/nrm2786 19851336 10.1038/nrm2786 PMC2834236

[42] Togo T, Steinhardt RA (2004) Nonmuscle myosin IIA and IIB have distinct functions in the exocytosis-dependent process of cell membrane repair. Mol Biol Cell 15: 688–695 doi:10.1091/mbc.E03-06-0430 14617807 10.1091/mbc.E03-06-0430 PMC329289

[43] Swailes NT, Colegrave M, Knight PJ, Peckham M (2006) Non-muscle myosins 2A and 2B drive changes in cell morphology that occur as myoblasts align and fuse. J Cell Sci 119: 3561–3570 doi:10.1242/jcs.03096 16895968 10.1242/jcs.03096

[44] Sakurai T, Fujita Y, Ohto E, Oguro A, Atomi Y (2005) The decrease of the cytoskeleton tubulin follows the decrease of the associating molecular chaperone alphaB-crystallin in unloaded soleus muscle atrophy without stretch. FASEB J 19: 1199–1201 doi:10.1096/fj.04-3060fje 15894563 10.1096/fj.04-3060fje

[45] Féasson L, Stockholm D, Freyssenet D, Richard I, Duguez S, et al (2002) Molecular adaptations of neuromuscular disease-associated proteins in response to eccentric exercise in human skeletal muscle. J Physiol (Lond) 543: 297–306. 12181300 10.1113/jphysiol.2002.018689 PMC2290467

[46] Mermelstein CS, Portilho DM, Medeiros RB, Matos AR, Einicker-Lamas M, et al (2005) Cholesterol depletion by methyl-beta-cyclodextrin enhances myoblast fusion and induces the formation of myotubes with disorganized nuclei. Cell Tissue Res 319: 289–297 doi:10.1007/s00441-004-1004-5 15549398 10.1007/s00441-004-1004-5

[47] Goudenege S, Dargelos E, Claverol S, Bonneu M, Cottin P, et al (2007) Comparative proteomic analysis of myotube caveolae after milli-calpain deregulation. Proteomics 7: 3289–3298 doi:10.1002/pmic.200700124 17849407 10.1002/pmic.200700124

[48] Volonte D, Peoples AJ, Galbiati F (2003) Modulation of myoblast fusion by caveolin-3 in dystrophic skeletal muscle cells: implications for Duchenne muscular dystrophy and limb-girdle muscular dystrophy-1C. Mol Biol Cell 14: 4075–4088 doi:10.1091/mbc.E03-03-0161 14517320 10.1091/mbc.E03-03-0161 PMC207001

[49] Hill MM, Bastiani M, Luetterforst R, Kirkham M, Kirkham A, et al (2008) PTRF-Cavin, a conserved cytoplasmic protein required for caveola formation and function. Cell 132: 113–124 doi:10.1016/j.cell.2007.11.042 18191225 10.1016/j.cell.2007.11.042 PMC2265257

[50] Ogata T, Ueyama T, Isodono K, Tagawa M, Takehara N, et al (2008) MURC, a muscle-restricted coiled-coil protein that modulates the Rho/ROCK pathway, induces cardiac dysfunction and conduction disturbance. Mol Cell Biol 28: 3424–3436 doi:10.1128/MCB.02186-07 18332105 10.1128/MCB.02186-07 PMC2423172

[51] Bastiani M, Liu L, Hill MM, Jedrychowski MP, Nixon SJ, et al (2009) MURC/Cavin-4 and cavin family members form tissue-specific caveolar complexes. J Cell Biol 185: 1259–1273 doi:10.1083/jcb.200903053 19546242 10.1083/jcb.200903053 PMC2712963

[52] Leroy B, Rosier C, Erculisse V, Leys N, Mergeay M, et al (2010) Differential proteomic analysis using isotope-coded protein-labeling strategies: comparison, improvements and application to simulated microgravity effect on Cupriavidus metallidurans CH34. Proteomics 10: 2281–2291 doi:10.1002/pmic.200900286 20391527 10.1002/pmic.200900286

[53] Elias JE, Haas W, Faherty BK, Gygi SP (2005) Comparative evaluation of mass spectrometry platforms used in large-scale proteomics investigations. Nat Methods 2: 667–675 doi:10.1038/nmeth785 16118637 10.1038/nmeth785

[54] Mastroleo F, Van Houdt R, Leroy B, Benotmane MA, Janssen A, et al (2009) Experimental design and environmental parameters affect Rhodospirillum rubrum S1H response to space flight. ISME J 3: 1402–1419 doi:10.1038/ismej.2009.74 19571896 10.1038/ismej.2009.74

[55] Shi Y, Elmets CA, Smith JW, Liu Y-T, Chen Y-R, et al (2007) Quantitative proteomes and in vivo secretomes of progressive and regressive UV-induced fibrosarcoma tumor cells: mimicking tumor microenvironment using a dermis-based cell-trapped system linked to tissue chamber. Proteomics 7: 4589–4600 doi:10.1002/pmic.200700425 18022937 10.1002/pmic.200700425

[56] Zhu C-H, Mouly V, Cooper RN, Mamchaoui K, Bigot A, et al (2007) Cellular senescence in human myoblasts is overcome by human telomerase reverse transcriptase and cyclin-dependent kinase 4: consequences in aging muscle and therapeutic strategies for muscular dystrophies. Aging Cell 6: 515–523 doi:10.1111/j.1474-9726.2007.00306.x 17559502 10.1111/j.1474-9726.2007.00306.x

[57] Oldfors A (2007) Hereditary myosin myopathies. Neuromuscul Disord 17: 355–367 doi:10.1016/j.nmd.2007.02.008 17434305 10.1016/j.nmd.2007.02.008

[58] Swailes NT, Colegrave M, Knight PJ, Peckham M (2006) Non-muscle myosins 2A and 2B drive changes in cell morphology that occur as myoblasts align and fuse. J Cell Sci 119: 3561–3570 doi:10.1242/jcs.03096 16895968 10.1242/jcs.03096

