# Supplementary figures and images for: FSHD Myotubes with Different Phenotypes Exhibit Distinct Proteomes
Source: PLoS One. 2012 Dec 18;7(12):e51865. doi: 10.1371/journal.pone.0051865 (PMC3525578; doi:10.1371/journal.pone.0051865)

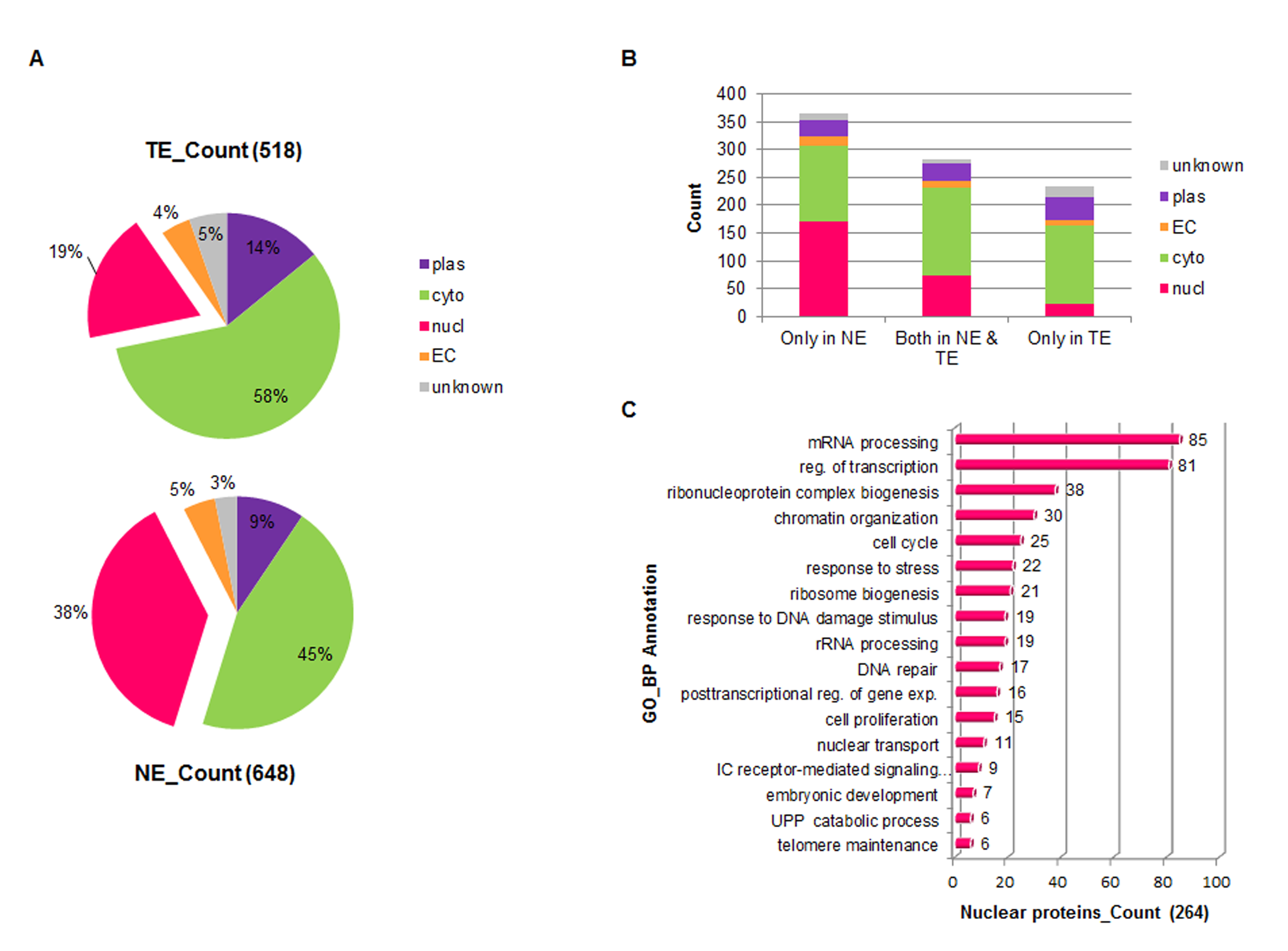

Supplement: Figure S1 — Comparison of protein composition in total (TE) and nuclear protein-enriched (NE) fractions of FSHD myotubes (analysis #P2 and P3). The analysis was conducted using TE and NE of FSHD myotubes (dFSHD12) 4 days after differentiation by 2DLC-MS/MS without ICPL labeling. (A) Subcellular classification of the detected proteins in TE and NE of FSHD myotubes. (B) Histograms comparing the number of detected proteins in TE and NE of FSHD myotubes and their subcellular localizations. (C) Functional classification of nuclear proteins detected in FSHD myotubes. The subcellular and functional classifications were conducted using ingenuity pathway analysis (IPA) or David database bioinformatics tools, respectively. (TIF) [file pone.0051865.s001.tif]

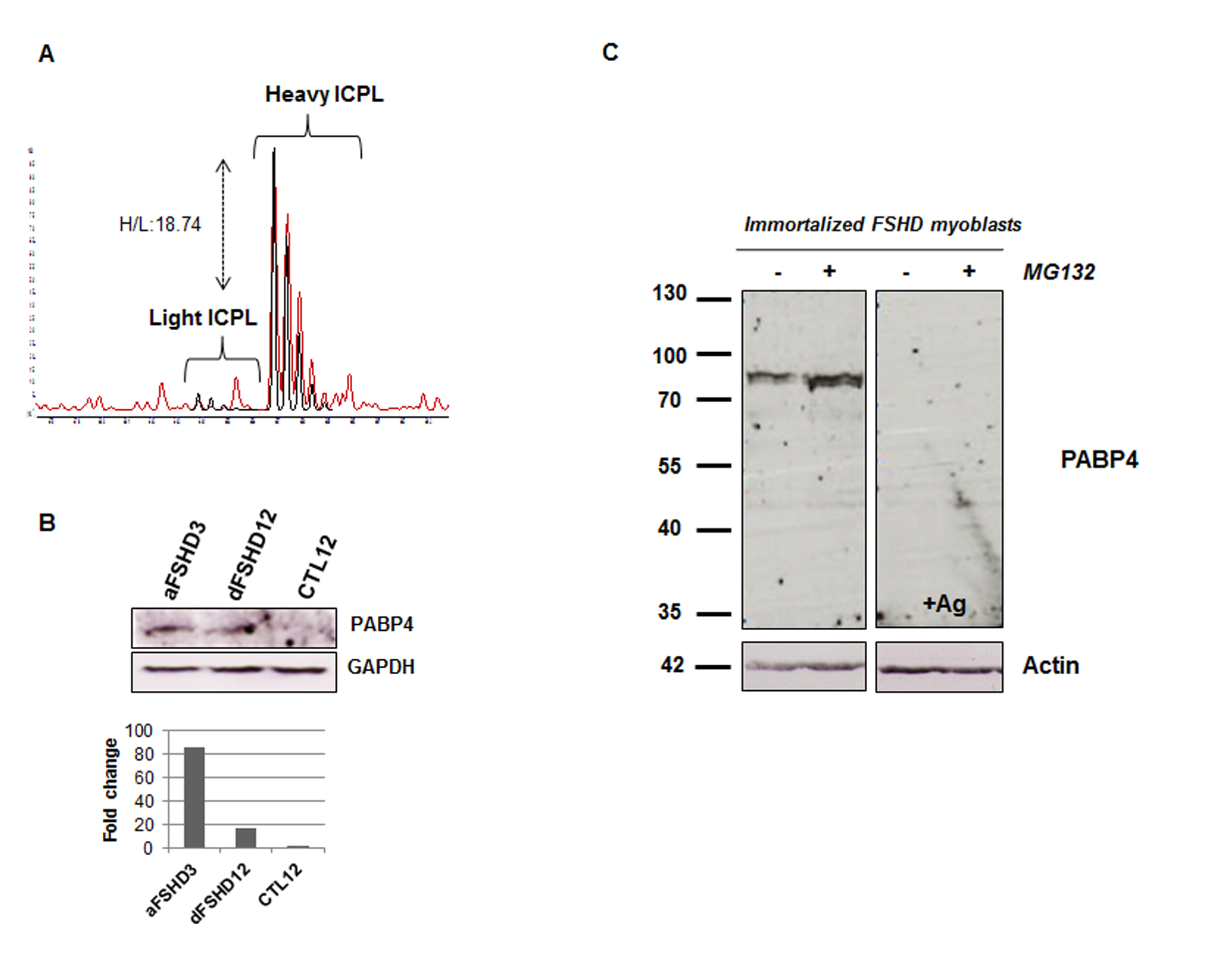

Supplement: Figure S2 — The polyA-binding protein 1/4 (PABP1/4) is up-regulated in FSHD myotubes (A) MS spectrum of the AHLTNQYMQ peptide that is common to PABP1 and PABP4 proteins. The graph represents the isotopic distribution corresponding to the FSHD peptide labeled with the heavy ICPL tag (right) and the control peptide labeled with the light ICPL tag (left). The H/L intensity ratio of 18.74 corresponds to the relative protein quantification. The theoretical and experimental spectra are indicated in red or black, respectively. (B) Western blot analysis of TE of atrophic (aFSHD3), disorganized (dFSHD12) and control (CTL12) myotubes using an antibody directed against PABP4 (Bethyl Laboratories). The bottom panel corresponds to the densitometry analysis. (C) Specificity of the anti-PABP4 antibody. Immortalized human myoblasts were kindly provided by Drs. G. Butler-Browne and V. Mouly (Institute of Myology, Paris). These lines were derived from a non-affected control (LHCN-M2) and were immortalized as described in [56]. They were cultivated and differentiated for 4 days, as described in [20]. Putative regulation by proteolytic degradation was evaluated by adding the proteasome inhibitor MG132 (25 µM, Sigma Aldrich) to the culture medium 5 h before the cells were harvested. Total cell protein extracts (20 µg, RIPA buffer) was separated by 12% SDS-PAGE, transferred to a nitrocellulose membrane and immunodetected with the anti-PABP4 antibody. A band at the expected MW for PAPB4 was detected, and this signal disappeared upon competition with a 5-fold excess of the antigenic peptide (+Ag, Bethyl Laboratories). The addition of MG132 slightly improved PABP4 detection. (TIF) [file pone.0051865.s002.tif]

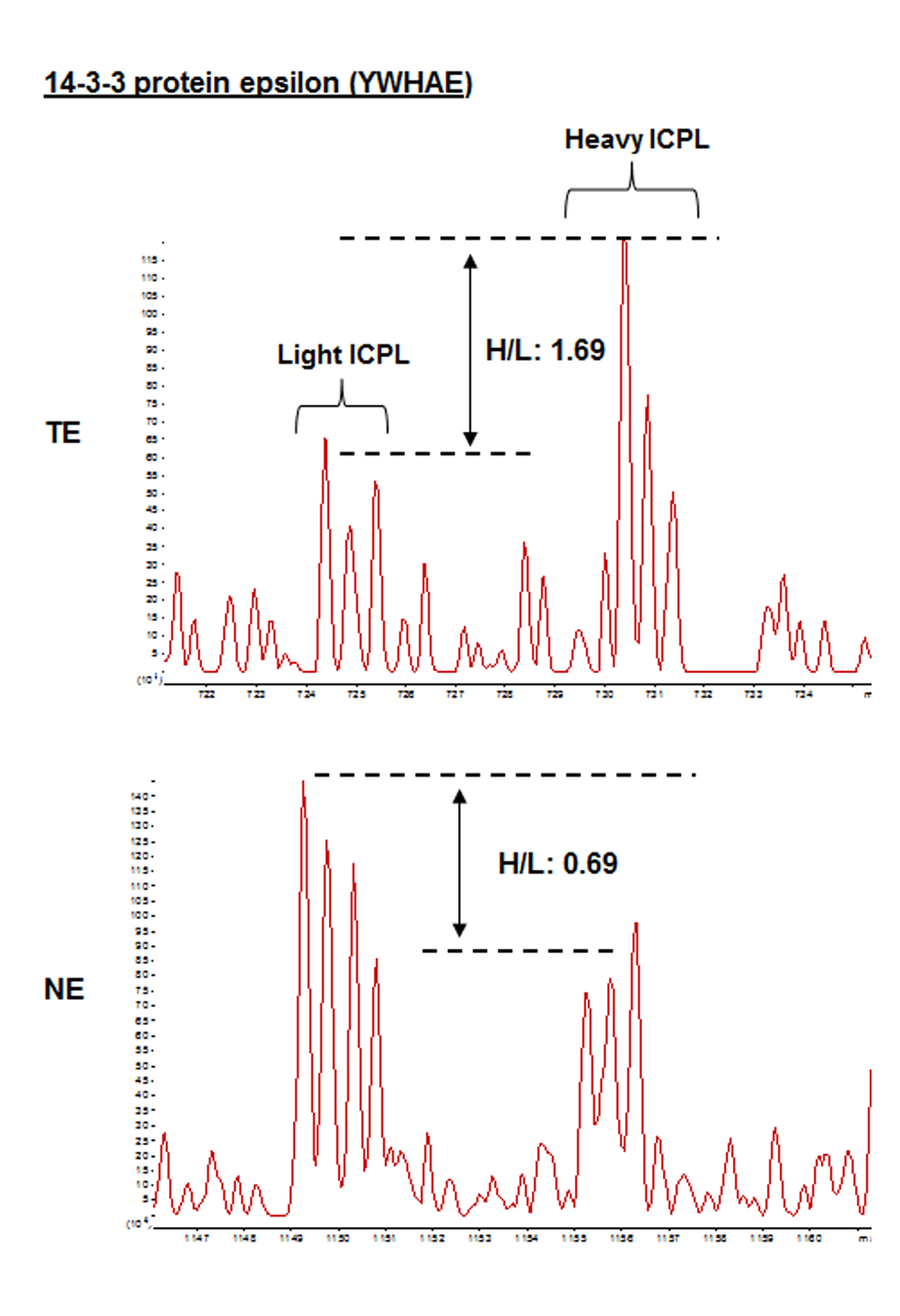

Supplement: Figure S3 — Changes in the 14-3-3 protein epsilon (YWHAE) intracellular distribution suggest a disruption of its nuclear-cytoplasmic shuttling in FSHD myotubes. Representative MS spectrum of the 14-3-3 protein epsilon peptide quantified by 2DLC-MS/MS in TE and NE of aFSHD3 myotubes. The graph represents the isotopic distribution corresponding to the FSHD peptide labeled with the heavy ICPL tag (right) and the control peptide labeled with the light ICPL tag (left). The indicated H/L intensity ratios correspond to the relative protein quantification. (TIF) [file pone.0051865.s003.tif]

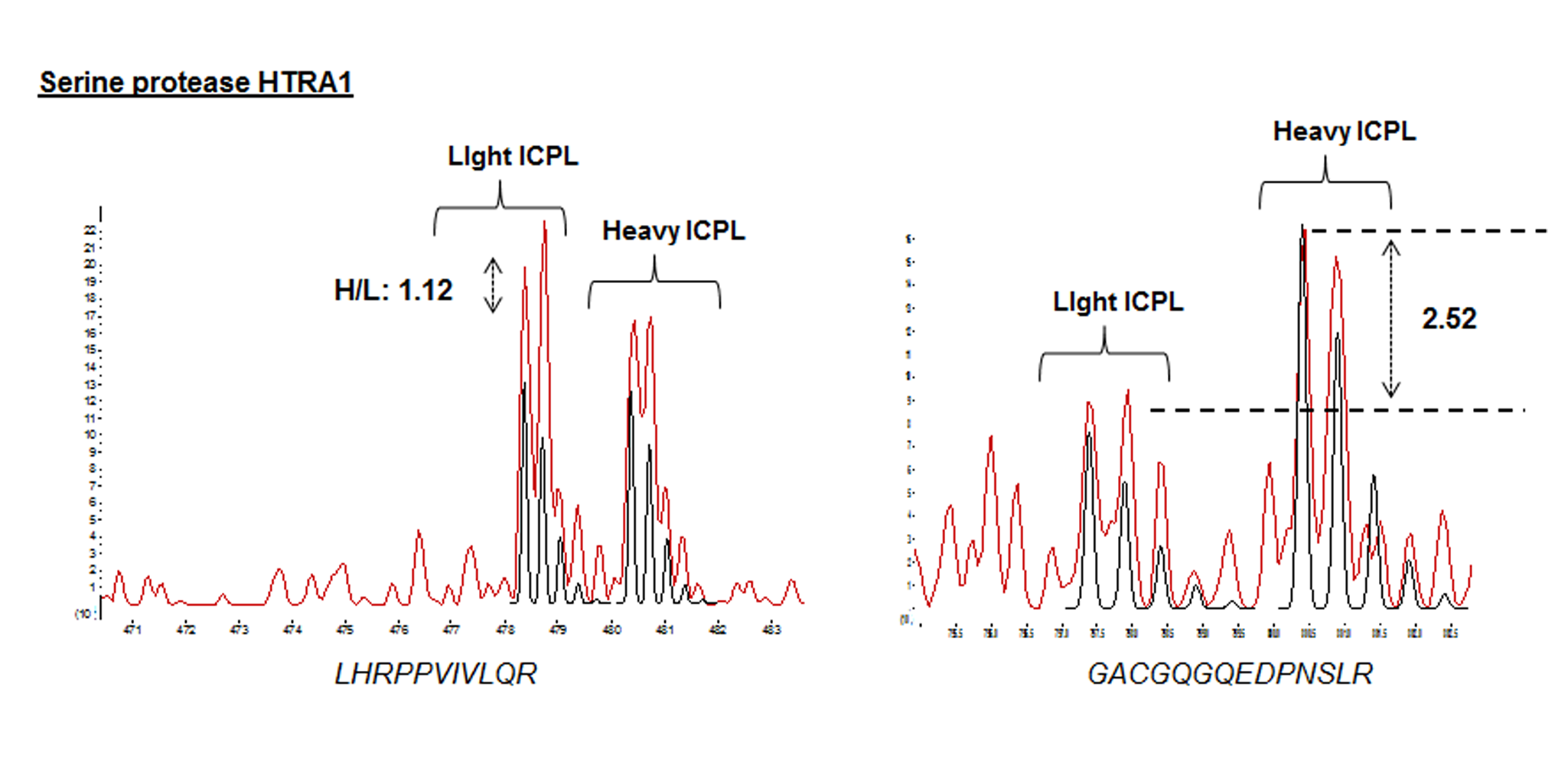

Supplement: Figure S4 — Quantification of the serine protease HTRA1 suggests the presence of two isoforms, and only one appears to have increased expression in atrophic FSHD myotubes. Representative MS spectra of HTRA1 peptides quantified by 2DLC-MS/MS in TE of aFSHD3 myotubes. The graph represents the isotopic distribution corresponding to the FSHD peptide labeled with the heavy ICPL tag (right) and the control peptide labeled with the light ICPL tag (left). The indicated H/L intensity ratios correspond to the relative protein quantification. (TIF) [file pone.0051865.s004.tif]
