# Supplementary material for: FSHD Myotubes with Different Phenotypes Exhibit Distinct Proteomes
Source: PLoS One. 2012 Dec 18;7(12):e51865. doi: 10.1371/journal.pone.0051865 (PMC3525578; doi:10.1371/journal.pone.0051865)
Supplement: Table S1 — Summary of published transcriptomic and proteomic studies on FSHD myoblasts and muscle biopsies. d5-7: 5 to 7 days of differentiation; d8: 8 days of differentiation. (DOCX) [file pone.0051865.s005.docx]

**Table S1. Summary of published transcriptomic and proteomic studies on FSHD myoblasts and muscle biopsies.**

| **References** | **Methods** | **Samples** | **Muscle types and comparisons** | **Functional categories of deregulated genes/proteins** |
| --- | --- | --- | --- | --- |
| **Tupler *et al.,* 1999** | **Transcriptomics** (mRNAs subtractive hybridization) | Biopsies | Deltoid | Global misregulation of gene expression in FSHD |
| **Winokur *et al.,* 2003** | **Transcriptomics** (Microarray) | Biopsies | Biceps (affected muscle) and deltoid (non-affected muscle) compared to normal muscle and other types of muscular dystrophy | FSHD-specific deregulated genes : involved in myogenic differentiation (targets of MYOD) and in response to oxidative stress |
| **Winokur *et al.,* 2003** | **Transcriptomics** (Microarray) | Myoblasts |  | Oxidative stress; extracellular matrix; cell cycle (p21) |
| **Laoudj-Chenivesse *et al.,* 2004** | **Proteomics** (2D gel and MALDI-TOF) | Biopsies | Deltoid,quadriceps, trapezius, subscapularis (healthy, DMD and FSHD muscles) | Mitochondrial metabolism and response to oxidative stress in affected and non-affected muscles |
| **Osborne *et al.,* 2006** | **Transcriptomics** (Microarray) | Biopsies | Quadriceps | Gene specifically upregulated in FSHD : role in vascular smooth muscle or endothelial cells |
| **Celegato *et al.,* 2006** | **Transcriptomics** (Microarray) and **proteomics** (2D gel and HPLC-ESI-MS/MS) | Biopsies | Deltoid of patients with variable number of KpnI repeats | Transition from fast-glycolytic to slow-oxidative phenotype in FSHD muscle; deficit in response to oxidative stress; disruption in the MyoD-dependent gene network |
| **Arashiro *et al.,* 2009** | **Transcriptomics** (Microarray) | Biopsies | Biceps and deltoid from affected, asymptomatic carrier and control individuals | **Asymptomatic muscles** : genes encoding chemokines; **affected muscles** : genes involved in the synthesis of GPI-linked proteins and histone acetylation |
| **Tsumagari *et al.,* 2011** | **Transcriptomics** (Microarray) | Undifferentiated and differentiated myoblasts (d5-7) | Moderately affected muscle | Dampening of normal myogenesis expression pattern : genes involved in muscle structure, mitochondrial function, stress response, signal transduction, RNA splicing (miRNA function); extra-cellular matrix and pro-inflammatory proteins |
| **Chely *et al.,* 2011** | **Transcriptomics** (Microarray) | Myotubes (d8) | FSHD1 vs FSHD2 | **FSHD1** : defect of cell cycle progression; **FSHD2** : defect in protein synthesis |
