# Supplementary material for: FSHD Myotubes with Different Phenotypes Exhibit Distinct Proteomes
Source: PLoS One. 2012 Dec 18;7(12):e51865. doi: 10.1371/journal.pone.0051865 (PMC3525578; doi:10.1371/journal.pone.0051865)
Supplement: Table S2 — Patient characteristics and 2DLC-MS/MS analysis. (A) Name of the FSHD cell line (code) as indicated in [27] (“line” refers to a myoblast population derived from a single biopsy; a: predominantly atrophic myotubes; d: predominantly disorganized myotubes); age and sex of the patient (M: male; F: female); number of D4Z4 units; site of the muscle biopsy [Q = quadriceps (vastus lateralis)]; score on the Brooke–Vignos scale defining the clinical status of upper and lower limb muscles, respectively, where high values define affected muscles and low values define non-affected muscles; predominant phenotype of the derived myotubes and MFI determined in [27] (myoblast fusion index: ratio between the nuclei present in myotubes versus the total number of nuclei in a given microscope field; the proportion of atrophied myotubes in a culture is inversely correlated with the MFI). (B) The following information is indicated for each 2DLC-MS/MS analysis: the FSHD and control myoblasts line that was compared, the differentiation stage (d4: 4 days; d6: 6 days), the extraction type (TE: total extracts; NE: fraction enriched in nuclear proteins), the ICPL procedure (regular or Post-digest), the SCX column (P: POROS10S, Dionex; B: Biobasic SCX, Thermo), the number of identified and quantified proteins and the total number of non-redundant identified peptides. (DOCX) [file pone.0051865.s006.docx]

**Table S2. Patient characteristics (A) and 2DLC-MS/MS analysis (B).**

| **A** | **Code^*^** | **Age** | **Sex** | **D4Z4 units** | **Muscle type (Brooke-Vignos scale)** | **Predominantly FSHD myotube phenotype (MFI %)** |
| --- | --- | --- | --- | --- | --- | --- |
|  | **aFSHD3** | 32 | F | 7 | Q(1-1) | Atrophic (37) |
|  | **dFSHD12** | 38 | F | 7 | Q(1-1) | Disorganized (60) |
|  | **a/dFSHD8** | 39 | M | 6 | Q(2-1) | Atrophic/Disorganized (53) |
|  | **CTL7** | 46 | M | ND | Q |  |
|  | **CTL12** | 35 | F | ND | Q |  |

*myoblasts previously characterized in Barro *et al.,* 2010

| **B** | **Analysis code** | **Myoblast lines** | **Differentiation stages** | **Protein extracts** | **ICPL methods** | **2DLC (SCX column types)** | **Number of identified proteins** | **Number of quantified proteins** | **Number of identified peptides** |
| --- | --- | --- | --- | --- | --- | --- | --- | --- | --- |
|  | **#P1** | a/dFSHD8 vs CTL7 | d6 | TE | Regular | P | 145 | 61 | 173 |
|  | **#P2** | dFSHD12 | d4 | TE | X | B | 518 | X | 1855 |
|  | **#P3** | dFSHD12 | d4 | NE | X | B | 648 | X | 3222 |
|  | **#dFSHD12_TE** | dFSHD12 vs CTL12 | d4 | TE | Post-digest | B | 268 | 195 | 1171 |
|  | **#dFSHD12_NE** | dFSHD12 vs CTL12 | d4 | NE | Post-digest | B | 292 | 215 | 939 |
|  | **#aFSHD3_TE** | aFSHD3 vs CTL12 | d4 | TE | Post-digest | B | 207 | 151 | 1302 |
|  | **#aFSHD3_NE** | aFSHD3 vs CTL12 | d4 | NE | Post-digest | B | 149 | 122 | 711 |
