# Supplementary material for: FSHD Myotubes with Different Phenotypes Exhibit Distinct Proteomes
Source: PLoS One. 2012 Dec 18;7(12):e51865. doi: 10.1371/journal.pone.0051865 (PMC3525578; doi:10.1371/journal.pone.0051865)
Supplement: Text S1 — Regular ICPL coupled to 2DLC-MS/MS (Analysis #P1). (DOCX) [file pone.0051865.s016.docx]

Protein extraction, ICPL and 2DLC-MS/MS procedures were optimized specifically for myotubes, which mainly contain cytoskeletal proteins. Four analyses were conducted with a gradual technical improvement (**Table S2B**), and the resulting preliminary data are presented bellow (**Texts S1-3**).

**Text S1. Regular ICPL coupled to 2DLC-MS/MS (Analysis #P1).**

In the first analysis, proteins from a total extract of FSHD myotubes (FSHD8, **Table S2A)** were labeled with the heavy ICPL tag, and proteins from healthy control myotubes (CTL7, **Table S2A**) were labeled with the light ICPL tag. The labeled proteins were digested with trypsin, and the resulting peptides were submitted to 2DLC-MS/MS (**Table S2B**, **Analysis #P1**). In these conditions, 146 proteins were identified, and 61 proteins were quantified. Most of the proteins were ribonucleoproteins (Gene Ontology GO0030529: 25%) and cytoskeletal proteins (Gene Ontology GO0005856: 25%). Despite a limited number of quantified proteins, the few changes observed reflect FSHD characteristics (**Fig. 1, see Text**). Despite the high sensitivity of the 2DLC-MS/MS approach, the main disadvantage of the proteomic analysis of myotubes is the abundance of contractile and other cytoskeletal proteins, which could mask the detection of lower abundance proteins and lead to limited protein quantification, as shown in this preliminary analysis.
