# Supplementary material for: FSHD Myotubes with Different Phenotypes Exhibit Distinct Proteomes
Source: PLoS One. 2012 Dec 18;7(12):e51865. doi: 10.1371/journal.pone.0051865 (PMC3525578; doi:10.1371/journal.pone.0051865)
Supplement: Text S2 — Myotube nuclear enrichment to improve protein detection (Analysis #P2-3). (DOCX) [file pone.0051865.s017.docx]

**Text S2. Myotube nuclear enrichment to improve protein detection (Analysis #P2-3).**

To decrease the abundance of cytoskeletal components, we have developed a nuclear enrichment procedure that does not involve detergents and is thus compatible with mass spectrometry (**See Materials and Methods section**). We have also used a high-resolution SCX column (Biobasic SCX, Thermo) for the ion exchange chromatography peptide separation step compared to the column used for analysis #P1 (SCX, POROS10S, Dionex). To assess the nuclear enrichment protocol and the efficacy of the new SCX column, we analyzed total extracts (**Table S2B: analysis #P2**) and a fraction enriched in nuclear proteins (**Table S2B: analysis #P3**) of dFSHD12 myotubes by 2DLC-MS/MS (**Table S6**). We observed an increase in the number of identified proteins, both in the total and nuclear extracts, compared to analysis #P1 (**Table S2B**). The number of identified proteins was also higher in the nuclear extracts (**Fig. S1A**, **B**), most likely because low-abundance proteins were not so frequently masked by cytoskeletal proteins, which had lower abundance in this fraction. Despite the remaining contamination of cytoplasmic proteins, we observed a content of 38% nuclear proteins (**Fig. S1A**) rather than the 19% fraction observed in the total extract. The nuclear proteins identified in analysis #P3 are mostly involved in mRNA processing and transcription regulation, but some are involved in chromatin organization, response to DNA damage and telomere maintenance (David database: Gene Ontology, **Fig. S1C**).

In summary, the nuclear enrichment allowed us to increase the number of quantified proteins by reducing the amount of obscuring cytoskeletal proteins. Structural proteins were still detectable because of their abundance or their interaction with nucleus components, or because of a secondary function in the nucleus as described for actin and myosin [1–4].

**References**

1. Dzijak R, Yildirim S, Kahle M, Novák P, Hnilicová J, et al. (2012) Specific nuclear localizing sequence directs two myosin isoforms to the cell nucleus in calmodulin-sensitive manner. PLoS ONE 7: e30529. doi:10.1371/journal.pone.0030529.

2. Chen M, Shen X (2007) Nuclear actin and actin-related proteins in chromatin dynamics. Curr Opin Cell Biol 19: 326–330. doi:10.1016/j.ceb.2007.04.009.

3. Ketema M, Wilhelmsen K, Kuikman I, Janssen H, Hodzic D, et al. (2007) Requirements for the localization of nesprin-3 at the nuclear envelope and its interaction with plectin. J Cell Sci 120: 3384–3394. doi:10.1242/jcs.014191.

4. Rando OJ, Zhao K, Crabtree GR (2000) Searching for a function for nuclear actin. Trends Cell Biol 10: 92–97.
