# Supplementary material for: FSHD Myotubes with Different Phenotypes Exhibit Distinct Proteomes
Source: PLoS One. 2012 Dec 18;7(12):e51865. doi: 10.1371/journal.pone.0051865 (PMC3525578; doi:10.1371/journal.pone.0051865)
Supplement: Text S3 — Post-digest ICPL to improve protein quantification in FSHD myotubes. (DOCX) [file pone.0051865.s018.docx]

**Text S3. Post-digest ICPL to improve protein quantification in FSHD myotubes.**

The regular Isotope Coded Protein Labeling (ICPL) is based on the labeling of free amino groups (N-terminal and lysines) of intact proteins by amine-specific reagents using different stable isotopes. The FSHD proteins were labeled with the heavy ICPL tag (H), and the control proteins were labeled with the light ICPL tag (L). In the post-digest ICPL version, proteins are digested with trypsin before ICPL labeling, thus ensuring the labeling of every peptide. Using this strategy, the number of identified and quantified proteins was increased in comparison with regular ICPL, as described in [1] (**Table S2B**).

**References**

1. Leroy B, Rosier C, Erculisse V, Leys N, Mergeay M, et al. (2010) Differential proteomic analysis using isotope-coded protein-labeling strategies: comparison, improvements and application to simulated microgravity effect on Cupriavidus metallidurans CH34. Proteomics 10: 2281–2291. doi:10.1002/pmic.200900286.
