# Supplementary material for: FSHD Myotubes with Different Phenotypes Exhibit Distinct Proteomes
Source: PLoS One. 2012 Dec 18;7(12):e51865. doi: 10.1371/journal.pone.0051865 (PMC3525578; doi:10.1371/journal.pone.0051865)
Supplement: Text S4 — Validation of the increased amount of PABP in FSHD myotubes. (DOCX) [file pone.0051865.s019.docx]

**Text S4. Validation of the increased amount of PABP in FSHD myotubes (Fig S2).**

We observed moderate perturbations of proteins involved in RNA processing such as hnRNP or splicing factors in FSHD primary myotubes (**Fig 2D**). However, the most dysregulated-protein in dFSHD12 myotubes (i.e., with an 18-fold upregulation) was polyA-binding protein (PABP) which plays various roles in mRNA processing. The detection of this protein was based on one peptide (AHLTNQYMQ) and was submitted to manual validation using the criteria defined in [1]. The corresponding MS spectrum (**Fig. S2A**) indicated a strong increase in FSHD myotube extract (heavy ICPL tag). Five PABP proteins have been described, one in the nucleus (PABP2), four in the cytoplasm (PABP1, 3-5), and the peptide detected here is common between PABP4 (iPABP) and PABP1. Both isoforms are highly similar at the protein level and bind polyA sequences with a similar affinity, which suggests that they may function similarly in the regulation of global mRNA translation and stability [2]. To validate the proteomic data, we prepared a Western blot with the total myotube extracts used in 2DLC-MS/MS and immunodetected PABP4 using an antibody directed against residues 525-575 (peptide competition**: Fig. S2C)**. This experiment confirmed PABP4 upregulation in FSHD myotubes (**Fig. S2B**).

The DUX4 dysregulation cascade included other genes involved in RNA processing [3], and this alteration could also affect cell differentiation. Moreover, FRG1, another FSHD candidate gene, was also found to be involved in multiple aspects of RNA biogenesis, including mRNA transport, cytoplasmic localization [4] and pre-messenger RNA splicing [5,6]. Thus, both DUX4 and FRG1 could affect mRNA processing in FSHD myotubes.

**References**

1. Leroy B, Rosier C, Erculisse V, Leys N, Mergeay M, et al. (2010) Differential proteomic analysis using isotope-coded protein-labeling strategies: comparison, improvements and application to simulated microgravity effect on Cupriavidus metallidurans CH34. Proteomics 10: 2281–2291. doi:10.1002/pmic.200900286.

2. Burgess HM, Richardson WA, Anderson RC, Salaun C, Graham SV, et al. (2011) Nuclear relocalisation of cytoplasmic poly(A)-binding proteins PABP1 and PABP4 in response to UV irradiation reveals mRNA-dependent export of metazoan PABPs. J Cell Sci 124: 3344–3355. doi:10.1242/jcs.087692.

3. Bosnakovski D, Xu Z, Gang EJ, Galindo CL, Liu M, et al. (2008) An isogenetic myoblast expression screen identifies DUX4-mediated FSHD-associated molecular pathologies. EMBO J 27: 2766–2779. doi:10.1038/emboj.2008.201.

4. Sun C-YJ, van Koningsbruggen S, Long SW, Straasheijm K, Klooster R, et al. (2011) Facioscapulohumeral muscular dystrophy region gene 1 is a dynamic RNA-associated and actin-bundling protein. J Mol Biol 411: 397–416. doi:10.1016/j.jmb.2011.06.014.

5. Gabellini D, D’Antona G, Moggio M, Prelle A, Zecca C, et al. (2006) Facioscapulohumeral muscular dystrophy in mice overexpressing FRG1. Nature 439: 973–977. doi:10.1038/nature04422.

6. van Koningsbruggen S, Straasheijm KR, Sterrenburg E, de Graaf N, Dauwerse HG, et al. (2007) FRG1P-mediated aggregation of proteins involved in pre-mRNA processing. Chromosoma 116: 53–64. doi:10.1007/s00412-006-0083-3.
